# Density of convex intersections and applications

**DOI:** 10.1098/rspa.2016.0919

**Published:** 2017-09-20

**Authors:** M. Hintermüller, C. N. Rautenberg, S. Rösel

**Affiliations:** 1 Department of Mathematics, Humboldt-Universität zu Berlin, Unter den Linden 6, 10099 Berlin, Germany; 2 Weierstrass Institute, Mohrenstrasse 39, 10117 Berlin, Germany

**Keywords:** density, convex constraints, variational inequalities, finite elements, image restoration, elasto-plasticity

## Abstract

In this paper, we address density properties of intersections of convex sets in several function spaces. Using the concept of *Γ*-convergence, it is shown in a general framework, how these density issues naturally arise from the regularization, discretization or dualization of constrained optimization problems and from perturbed variational inequalities. A variety of density results (and counterexamples) for pointwise constraints in Sobolev spaces are presented and the corresponding regularity requirements on the upper bound are identified. The results are further discussed in the context of finite-element discretizations of sets associated with convex constraints. Finally, two applications are provided, which include elasto-plasticity and image restoration problems.

## 1. Introduction

Convex constraint sets *K* as subsets of an infinite-dimensional Banach space *X* are common to many fields in mathematics such as calculus of variations, variational inequalities and control theory. Such constraints are induced by physical limitations of control and/or state variables, but also emerge through Fenchel dualization of convex problems; e.g. [1–3] for fundamental concepts in variational analysis. In this vein, given a set of functions satisfying an arbitrary convex constraint, density properties of more regular functions satisfying the same restriction are of utmost importance. In abstract terms, given some dense subspace *Y* of *X*, the central point of interest is whether the closure property

1.1 $${\bar{K(Y)}}^{X}=K,$$

with *K*(*Y*)={*u*∈*Y* :*u*∈*K*}=*K*∩*Y* , is fulfilled, and how this problem is intimately linked to the solution of constrained optimization and variational inequality problems.

In the literature, problems of dense intersections appear in connection with the discretization of variational inequality problems in Sobolev spaces and the convergence analysis for finite-element methods under minimal regularity (e.g. [4–6]). Moreover, the limiting behaviour of singular perturbations of elliptic variational inequalities can be traced back to the density issue (see [7] and references therein). This also pertains to the deduction of a vanishing viscosity limit for hyperbolic variational inequalities with an obstacle constraint [8]. In the context of plasticity problems, certain density properties represent an important step towards the determination of appropriate relaxed formulations (cf. [9,10]). However, to the best of our knowledge, the investigation of problem (1.1) is restricted to special cases and the literature still lacks a general and systematic treatment of the density issue.

To motivate the study of the abstract problem (1.1), §2 provides a novel unifying framework for various perturbation approaches to non-smooth constrained optimization and variational inequality problems. The general setting includes regularization, Galerkin approximation and singular perturbations, and, most remarkably, it allows to reduce the study of the corresponding limit problems for a wide range of practically relevant perturbations to the study of the density property (1.1). In particular, we prove that the dense intersection (1.1) is a necessary and sufficient stability condition for the retrieval of the original problem in the (joint) limit of vanishing regularization and/or discretization parameters.

Starting from §3 we focus on the setting where *X*=*X*(*Ω*) is a ($R^{d}$-valued) vector space of functions over a bounded domain *Ω* of $R^{N}$ and *K*=*K*(*X*) denotes the subset of elements in *X*(*Ω*) bounded pointwise by a prescribed measurable function α:Ω→R∪{+∞}, i.e.

K(X(Ω))={w∈X(Ω):|w(x)|≤α(x) a.e. (almost everywhere) in Ω},

with |⋅| denoting an $R^{d}$-norm. Particularly in this part, *X*(*Ω*) refers to a Lebesgue or Sobolev space and *Y* =*Y* (*Ω*) refers to the space of continuous or infinitely differentiable functions up to the boundary. We also use the notation *K*(*X*(*Ω*);|⋅|) whenever it is necessary to make the dependence on the norm |⋅| explicit. Despite the fact that a small number of specific density results for very regular bounds *α* are available [4,9,11], a systematic investigation of density properties in terms of the regularity of *α* seems not available in the literature.

In order to close this gap, we prove new density results for continuous obstacles (§4), and we also consider different classes of discontinuous obstacles. In fact, in §4a, the density issue is studied in the context of the regularity of the obstacle as a Sobolev function. More precisely, we prove that results of the type (1.1) cannot be expected if the obstacle is just a Sobolev function by providing a counterexample. The density results are then proved to be valid even for certain classes of lower semicontinuous obstacles; see §4b,c. Subsequently, in §4d, a different approach is considered for obstacles that originate from the solution of a partial differential equation (PDE).

In §5, we focus on the Mosco convergence of finite-element discretized convex sets, which, in general, is a delicate matter, and only a limited number of results for more regular obstacles are known (e.g. [4,5]). In this respect, the construction of a recovery sequence essentially reduces to the verification of density properties of the type (1.1). Making use of the density results provided by the preceding sections, we prove several new Mosco convergence results in the Hilbert spaces *L*^2^,*H*^1^ and *H*(div) for different types of finite-element discretizations of *K*, even for discontinuous obstacles *α*. The results are extended to a more general constraint setting involving pointwise restrictions on partial derivatives. We conclude the paper by presenting two important applications that further highlight the paramount significance of dense intersections. First, we consider the regularization of an elasto-plastic contact problem, where the closure property turns out to be fundamental for the efficient solution by a semismooth Newton method. Secondly, we discuss an example from total variation-based image restoration with a distributed non-smooth regularization parameter. Here, the density property arises as an essential condition for the equivalent reformulation of the problem in the Hilbert space *H*(div) by means of Fenchel duality.

## 2. Motivation

### (a) Optimization with convex constraints

In many variational problems, one seeks the solution in a given convex, closed and non-empty subset *K* of an infinite-dimensional Banach space (*X*,∥.∥). To illustrate the problem, let us consider the following abstract class of optimization problems:

2.1 $$\begin{array}{rl} \inf\; & F(u),\;\text{over }u\in X,\\ \text{s.t.}\; & u\in K. \end{array}\}$$

We assume that F:X→R is continuous, coercive and sequentially weakly lower semicontinuous, but not necessarily convex. Thus, problem (2.1) admits a solution provided *X* is reflexive. The problem class (2.1) is ubiquitous, encompassing numerous fields, such as the variational form of PDEs, variational inequality problems of potential type, optimal control of PDEs with constraints on the state and/or control, and many other. The analysis of (2.1) and the design of suitable solution algorithms often involve the general concepts of perturbation or dualization methods comprising regularization, penalization or discretization approaches or possibly a combination of the latter (e.g. [1–5] and references therein). The central result of this section is that the stability of (2.1) with respect to a large class of perturbations can be characterized by the closure property (1.1), i.e.

$${\bar{K(Y)}}^{X}=K,$$

where *Y* is some dense subspace of *X* (in the norm topology of *X*), and *K*(*Y*) is given by

K(Y)={u∈Y:u∈K}=K∩Y.

In what follows, we will identify a very general class of perturbations for which the stability analysis effectively reduces to the study of the density property (1.1).

#### (i) A class of quasi-monotone perturbations

To subsume as many of the above-mentioned methods as possible, we consider the sequence of perturbed problems

2.2 $$\inf\;F(u)+R_{n}(u),\;\text{over }u\in X,$$

defined by a given sequence of functions

$$R_{n}:X\to R\cup \{+\infty \},\;n\in N,$$

that are perturbations of the indicator function $i_{K}:X\to R\cup \{+\infty \}$ in the following sense: there exist functions ${\underline{R}}_{n}:X\to R\cup \{+\infty \}$ and ${\bar{R}}_{n}:X\to R\cup \{+\infty \}$ such that

$$0\leq {\underline{R}}_{n}\leq R_{n}\leq {\bar{R}}_{n}\;\forall n\in N,$$

having the additional properties

2.3 $$\begin{array}{rl} & {\underline{R}}_{n}\leq {\underline{R}}_{n+1}\;\forall n\in N,\;\lim_{n\to +\infty }{\underline{R}}_{n}(u)=i_{K}(u)\;\forall u\in X\\ \text{and}\;\; & {\underline{R}}_{n}\text{ is sequentially weakly lower semicontinuous}\;\forall n\in N \end{array}\}$$

and

2.4 $${\bar{R}}_{n}\geq {\bar{R}}_{n+1},\;\forall n\in N,\;\lim_{n\to +\infty }{\bar{R}}_{n}(u)=i_{K\cap Y}(u)\;\forall u\in X.$$

We call mappings (*R*_*n*_) that share the above features *quasi-monotone perturbations of the indicator function* *i*_*K*_ *with respect to the* (*dense*) *subspace* *Y* . Note that no additional assumptions are made on (*R*_*n*_) itself.

The first main result of this section states that the dense intersection property implies the stability of *any* quasi-monotone perturbation scheme. The proof is deferred to appendix A.

**Theorem 2.1 (Sufficient condition)**

Let the Banach space X be reflexive or assume that the dual space X* *is separable. For a closed, convex and non-empty set K⊂X, let (R*_*n*_*) be a sequence of quasi-monotone perturbations of i*_*K*_ *with respect to the dense subspace Y according to (2.2). If the density property (1.1) holds true, then F+i*_*K*_ *is the Γ-limit of (F+R*_*n*_*) in both, the weak and strong topology.*

Under the assumptions of theorem 2.1, one may infer that, provided each problem (2.2) admits a global minimizer *u*_*n*_, each weak cluster point of the sequence of minimizers (*u*_*n*_) is a global minimizer of (2.1); see [12] for an introduction to *Γ*-convergence. At the end of this section, it is further clarified that theorem 2.1 is sharp in the sense that the stability result in general fails if (1.1) does not hold. We also remark that in case the (sequential) weak and strong *Γ*-limits coincide, one usually uses the notion Mosco convergence.

In the following, we present a selection of approximation methods that fit into the general class of perturbations defined by (2.2), which bear high practical relevance. In favour of generality, we do not leave the abstract setting.

**Example 2.2 (Tikhonov regularization)**

Let (*Y*,∥…∥_*Y*_) be a Banach space which is densely and continuously embedded into *X*. For a sequence of positive non-decreasing parameters (*γ*_*n*_) with $\gamma_{n}\to +\infty$ and fixed *α*>0, consider in (2.2) the Tikhonov regularization

2.5 $$R_{n}(u)=i_{K}(u)+\frac{1}{2\gamma_{n}}∥u{∥}_{Y}^{\alpha },$$

where it is understood that $R_{n}(u)=+\infty$ if *u*∉*Y* . In fact, set ${\underline{R}}_{n}:=i_{K}$ for all n∈N and ${\bar{R}}_{n}:=R_{n}$. Obviously, (2.3) and (2.4) are satisfied such that (*R*_*n*_) fits into the context of quasi-monotone perturbations according to (2.2).

**Example 2.3 (Conforming discretization)**

Let *X* be a separable Banach space. Suppose (2.1) is approximated by a Galerkin approach using nested and conforming finite-dimensional subspaces *X*_*n*_, i.e. *X*_*n*_⊂*X* and *X*_*n*_⊂*X*_*n*+1_ for all n∈N, such that the Galerkin approximation property

$${\bar{\underset{n\in N}{⋃}X_{n}}}^{X}=X$$

is fulfilled. The resulting discrete counterpart of problem (2.1) is given by (2.2) with *R*_*n*_(*u*)=*i*_*K*∩*X*_*n*__. Setting ${\underline{R}}_{n}=i_{K}$, (2.3) is clearly fulfilled. Define $Y=\underset{n\in N}{⋃}X_{n}$, then (2.4) is fulfilled with ${\bar{R}}_{n}=R_{n}$.

**Example 2.4 (Combined Moreau–Yosida/Tikhonov regularization)**

Let *X* be a Hilbert space and (*Y*,∥…∥_*Y*_) be a Banach space that is densely and continuously embedded into *X*. For two sequences of positive non-decreasing parameters (*γ*_*n*_),(*γ*_*n*_′) with $\gamma_{n},\gamma_{n}^{\prime }\to +\infty$ and fixed *α*>0, consider the simultaneous Moreau–Yosida and Tikhonov regularization

2.6 $$R_{n}(u)=\frac{\gamma_{n}}{2}\underset{v\in K}{\inf}∥u-v{∥}^{2}+\frac{1}{2\gamma_{n}^{\prime }}∥u{∥}_{Y}^{\alpha },$$

with *α*>0 fixed, where it is understood that $R_{n}(u)=+\infty$ if *u*∉*Y* . Setting ${\underline{R}}_{n}(u)=(\gamma_{n}/2)\underset{v\in K}{\inf}{∥u-v∥}^{2}$, standard properties of the Moreau–Yosida regularization ensure that ${\underline{R}}_{n}$ satisfies (2.3) (e.g. [2], Prop. 17.2.1). Defining ${\bar{R}}_{n}(u)=i_{K}(u)+(1/2\gamma_{n}^{\prime })∥u{∥}_{Y}^{\alpha }$, (2.4) is verified as in the previous example.

**Example 2.5 (Conforming discretization and Moreau–Yosida regularization)**

Let *X* be a separable Hilbert space and (*γ*_*n*_) a sequence of positive non-decreasing parameters converging to +∞. The combination of regularization and discretization leads to the definition

2.7 $$R_{n}(u)=\frac{\gamma_{n}}{2}\underset{v\in K}{\inf}∥u-v{∥}^{2}+i_{X_{n}}(u),$$

where the sequence of spaces (*X*_*n*_) is defined as in example 2.3. Setting ${\underline{R}}_{n}(u)=(\gamma_{n}/2)\underset{v\in K}{\inf}∥u-v{∥}^{2}$ and ${\bar{R}}_{n}(u)=i_{K\cap X_{n}}(u)$, (2.3) and (2.4) are fulfilled with $Y=\underset{n\in N}{⋃}X_{n}$ and the framework of (2.2) applies.

Consequently, each of these perturbations is stable with respect to (2.1) provided the density result (1.1) is satisfied. It should also be emphasized that these examples only represent an assorted variety of perturbations that fit into the problem class (2.2).

Moreover, the density property (1.1) is also a necessary condition for the stability of perturbation schemes in the following sense: first, the *Γ*-limit of the approximation schemes defined in examples 2.2 and 2.3 can be calculated using similar arguments as in the proof of theorem 2.1. In fact, under the same conditions on *X*, one obtains $F+i_{\bar{K\cap Y}}$ as the weak and strong *Γ*-limit in both cases. Secondly, in the combined approaches of examples 2.4 and 2.5, theorem 2.1 guarantees that *F*+*i*_*K*_ is obtained as the weak-strong *Γ*-limit for *any* coupling of regularization parameter pairs [*γ*_*n*_,*γ*_*n*_′] and [*X*_*n*_,*γ*_*n*_], respectively. Let us put this statement into a perspective by means of the combined Galerkin/Moreau–Yosida approach (example 2.5). In this case, it is possible to prove the existence of a suitable combination of *n* and *γ*_*n*_ to recover *F*+*i*_*K*_ in the *Γ*-limit without resorting to the density property (1.1), see [13], Prop. 2.4.6. However, the proof is non-constructive and thus not immediately useful for the design of a stable numerical algorithm. On the other hand, if (1.1) is violated, the *Γ*-convergence to the original problem (2.1) cannot be guaranteed independently from the choice of the regularization/discretization parameter pair. In fact, the following result, which we prove in appendix A, holds true.

**Proposition 2.6 (Necessary condition)**

*Consider example 2.5 with the corresponding definitions of* *Y* *and* (*R*_*n*_). *Further suppose that* $\bar{K\cap Y}⊊K$. *Then for all* $x\in K\setminus \bar{K\cap Y}$ *there exists a strictly increasing sequence* (*γ*_*n*_) *with* $\gamma_{n}\to \infty$ *such that*

$$F(y_{n})+R_{n}(y_{n})↛F(x),$$

*for all* (*y*_*n*_)⊂*X* *with* *y*_*n*_→*x*, *i.e. there exists no recovery sequence at* *x* *in the norm topology*.

The analogous statement is valid in the case of combined Moreau–Yosida/Tikhonov regularizations given a fixed sequence (*γ*_*n*_′); cf. example 2.4. In conclusion, theorem 2.1 is sharp with respect to condition (1.1) in the sense of proposition 2.6 and the preceding discussion.

### (b) Elliptic variational inequalities

The density of convex intersections of the type (1.1) is also of fundamental importance for the analysis of perturbations of variational inequalities. Assuming *X* to be a Hilbert space and *K*⊂*X* non-empty, closed and convex, we consider the general variational inequality problem of the first kind,

2.8 $$\text{find }u\in X:\;\langle Au,v-u\rangle +i_{K}(v)-i_{K}(u)\geq \langle l,v-u\rangle ,\;\forall v\in X;$$

e.g. [7,14] for an introduction. Here, *l*∈X* is a linear, bounded operator and *A*:*X*→X* denotes a, in general, nonlinear operator on *X*. We further assume *A* to be Lipschitz continuous and strongly monotone, i.e. there exists *κ*>0 with

$$\langle Av-Au,v-u\rangle \geq \kappa ∥v-u{∥}^{2},\;\forall u,v\in X.$$

In the following, we investigate three main classes of perturbations of (2.8) and their relation to the density properties of convex intersections.

#### (i) Quasi-monotone perturbation

Consider the perturbed variational inequality problem,

2.9 $$\text{find }u_{n}\in X:\;\langle A_{n}u_{n},v-u_{n}\rangle +R_{n}(v)-R_{n}(u_{n})\geq \langle l_{n},v-u_{n}\rangle ,\;\forall v\in X,$$

where *A*_*n*_ and *l*_*n*_ are appropriate perturbations of *A* and *l*, respectively, and (*R*_*n*_) is a quasi-monotone perturbation of *i*_*K*_ with respect to a dense subspace *Y* of *X*. The stability of the approximation scheme (2.9) hinges on the density property (1.1). In fact, if the latter condition is fulfilled, then the sequence (*R*_*n*_) Mosco converges to *i*_*K*_ provided ${\underline{R}}_{n}$ is weakly lower semicontinuous. Under mild assumptions on (*A*_*n*_) and (*l*_*n*_) one may then invoke known stability results, cf. [7, p.99, 15], to conclude the consistency of the perturbation scheme with respect to the limit problem (2.8).

#### (ii) Galerkin approximation of variational inequalities

In general, finite-dimensional approximations of *K* are neither conforming nor nested as it was the case in examples 2.3 and 2.5, where *K* was ‘discretized’ by *K*∩*X*_*n*_, which is numerically realizable only in special cases. Instead, it is often more favourable to consider non-nested approximations *K*_*n*_⊂*X*_*n*_ that may contain infeasible elements, such that *K*_*n*_⊂*K* does not hold true in general [4,5]. As a result, the finite-dimensional variational inequality problems,

2.10 $$\text{find }u_{n}\in X_{n}:\;\langle A_{n}u_{n},v-u_{n}\rangle +i_{K_{n}}(v)-i_{K_{n}}(u_{n})\geq \langle l_{n},v-u_{n}\rangle ,\;\forall v\in X_{n},$$

do not fit into the framework of (2.9). Again, under mild assumptions on (*A*_*n*_) and (*l*_*n*_), the Mosco convergence of (*K*_*n*_) to *K* ensures that the approximation (2.10) is stable with respect to the limit problem (2.8). However, Mosco convergence requires the existence of a recovery sequence (see definition 4.5) for any element *u*∈*K*. To construct this sequence in the context of finite-element methods, one may use an interpolation procedure which typically is only defined on the (supposedly) dense subset *K*∩*Y* of *K*, where $Y=C^{k}(\bar{\Omega })$ for some $k\in N_{0}$ (cf. [4], II, Theorem 2.3 and §5). This leads again to problem (1.1).

#### (iii) Singular perturbations

The closure property (1.1) also plays a role in the limiting behaviour of singular perturbations. In fact, let *A*_1_:*Y* →*Y* * be a Lipschitz continuous and strongly monotone operator on a Hilbert space (*Y*,∥…∥_*Y*_) that embeds densely and continuously into *X*. For a sequence of regularization parameters (*γ*_*n*_) with $\gamma_{n}\to +\infty$ consider the perturbed problems,

2.11 $$\text{find }u_{n}\in K\cap Y:\;\left\langle \left(A+\frac{1}{\gamma_{n}}A_{1}\right)u_{n},v-u_{n}\right\rangle \geq \langle l,v-u_{n}\rangle ,\;\forall v\in K\cap Y.$$

Observe that problem (2.11) admits a unique solution *u*_*n*_∈*K*∩*Y* provided that *K*∩*Y* is closed in *Y* . The appropriate limit problem is then given by

2.12 $$\text{find }u\in {\bar{K\cap Y}}^{X}:\;\langle Au,v-u\rangle \geq \langle l,v-u\rangle ,\;\forall v\in {\bar{K\cap Y}}^{X}.$$

Note that (2.12) corresponds to the initial variational inequality problem if the density property (1.1) holds true. In this case, the sequence (*u*_*n*_) converges strongly in *X* to the solution of (2.8). Here, the assumptions on *A*_1_ may be alleviated. This type of application also plays a role in the analysis and the design of algorithms for hyperbolic variational inequalities through the vanishing viscosity approach. For details, [7, section 4.9, 8] may be consulted.

## 3. Density results for continuous obstacles

We first fix some notation. In this section, $\Omega \subset R^{N}$ denotes a bounded Lipschitz domain. The space of functions that are restrictions to *Ω* of smooth functions with compact support on $R^{N}$ is denoted by $D(\bar{\Omega })$,

$$D(\bar{\Omega })=\{\varphi {|}_{\Omega }:\varphi \in C_{c}^{\infty }(R^{N})\}.$$

The standard Lebesgue and Sobolev spaces over *Ω* are denoted by *L*^*p*^(*Ω*),*W*^1,*p*^(*Ω*) and $W_{0}^{1,p}(\Omega )$, and we also employ the spaces

$$H(\mathrm{div};\Omega )=\{u\in L^{2}(\Omega ;R^{N}):\mathrm{div}u\in L^{2}(\Omega )\}$$

and

$$H_{0}(\mathrm{div};\Omega )={\bar{C_{c}^{\infty }(\Omega ;R^{N})}}^{H(\mathrm{div};\Omega )}=\{u\in H(\mathrm{div};\Omega ):u\cdot \nu =0\text{ on }\partial \Omega \}.$$

In the recent paper [11], it has been shown that for any $\alpha \in C(\bar{\Omega })$ with

3.1 $$\underset{x\in \Omega }{\mathrm{ess}\;\inf}\alpha (x)>0,$$

the following density result for the spaces $X(\Omega )\in \{L^{p}{\left(\Omega \right)}^{d},W_{0}^{1,p}{\left(\Omega \right)}^{d},H_{0}(\mathrm{div};\Omega )\}$, and 1≤p<+∞, holds true:

3.2 $${\bar{K(C_{c}^{\infty }{\left(\Omega \right)}^{d})}}^{X(\Omega )}=K(X(\Omega )),$$

where the constraint set *K*(*X*(*Ω*)) with respect to a given subspace

$$X(\Omega )\subset L^{1}{\left(\Omega \right)}^{d}$$

is defined by a pointwise constraint on an arbitrary norm |⋅| on $R^{d}$, i.e.

3.3 K(X(Ω)):={w∈X(Ω):|w(x)|≤α(x) a.e. in Ω}.

Here, α:Ω→R∪{+∞} is a given non-negative Lebesgue measurable function. It is further understood that *d*=*N* in (3.2) if *X*(*Ω*)=*H*_0_(div;*Ω*).

When considering the case *X*=*W*^1,*p*^ instead of $W_{0}^{1,p}$ in (3.2), the choice of the approximating sequence from [11], Theorem 1, which relies on the trivial extension of Sobolev functions, fails. As a result, a different extension operator has to be employed.

**Theorem 3.1**

*Let* $\alpha \in C(\bar{\Omega })$ *fulfil (3.1) and* 1≤p<+∞*. Then it holds that
*

3.4 $${\bar{K(D{\left(\bar{\Omega }\right)}^{d})}}^{W^{1,p}{\left(\Omega \right)}^{d}}=K(W^{1,p}{\left(\Omega \right)}^{d}),$$

*i.e.* $K(D{\left(\bar{\Omega }\right)}^{d})$ *is dense in K(W*^1,*p*^*(Ω)*^*d*^*) with respect to the norm topology in W*^1,*p*^*(Ω)*^*d*^.

**Proof.**

Let *w*∈*K*(*W*^1,*p*^(*Ω*)^*d*^). Since *Ω* is a bounded Lipschitz domain we may extend *w* to a function in $W^{1,p}{\left(R^{N}\right)}^{d}$ using for each component the extension-by-reflection operator. The resulting operator

3.5 $$E:W^{1,p}{\left(\Omega \right)}^{d}\to W^{1,p}{\left(R^{N}\right)}^{d}$$

has the properties *Ew*|_*Ω*_=*w* for all *w*∈*W*^1,*p*^(*Ω*)^*d*^ and $E\in L(W^{1,p}{\left(\Omega \right)}^{d},W^{1,p}{\left(R^{N}\right)}^{d})$; see, for instance, [16]. Since *E* is obtained by a partition of unity argument using local reflection with respect to the Lipschitz graphs into which ∂*Ω* can be decomposed, the property |*w*(*x*)|≤*α*(*x*) in *Ω* is preserved by the extension in that

3.6 $$|(Ew)(x)|\leq E_{C(\bar{\Omega })}\alpha (x),\;\text{ a.e. }x\in R^{N},$$

where $E_{C(\bar{\Omega })}:C(\bar{\Omega })\to C(R^{N})$ denotes the application of the extension by reflection procedure to bounded uniformly continuous functions, i.e. $(E_{C(\bar{\Omega })}\alpha ){|}_{\Omega }=\alpha$. Further inspecting the construction of *E*, it may also be observed that the support of *Ew* is compactly contained in $R^{N}$. Analogously, we obtain $E_{C(\bar{\Omega })}\alpha \in C_{c}(R^{N})$. For a sequence (*ρ*_*n*_) of smooth mollifiers

3.7 $$\rho_{n}(x)=n^{N}\rho (nx),$$

where

$$\rho \in D(R^{N}),\;\rho \geq 0,\;\rho (x)=0\;\text{if }|x|\geq 1,\;\int_{\Omega }\rho \;dx=1,$$

we define the approximating sequence *S*_*n*_(*w*,*Ω*) to *w* by

3.8 $$S_{n}(w,\Omega )(x):=(\rho_{n}*Ew)(x)=\int_{R^{N}}Ew(y)\rho_{n}(x-y)\;dy,\;x\in R^{N}.$$

It is well known that

3.9 $$S_{n}(w,\Omega ){|}_{\Omega }\to w\text{ in }W^{1,p}{\left(\Omega \right)}^{d}\;\text{as }n\to \infty ,$$

and, since *Ew* has compact support in $R^{N}$, it holds that $S_{n}(w,\Omega ){|}_{\Omega }\in D{\left(\bar{\Omega }\right)}^{d}$. In order to achieve feasibility, we use the scaling sequence

$$\beta_{n}:={\left(1+\frac{\underset{x\in R^{N}}{\sup}|\alpha_{n}(x)-E_{C(\bar{\Omega })}\alpha (x)|}{\min_{x\in \bar{\Omega }}\alpha (x)}\right)}^{-1},$$

where $\alpha_{n}(x):=((E_{C(\bar{\Omega })}\alpha )*\rho_{n})(x),\;x\in R^{N}$. Since $E_{C(\bar{\Omega })}\alpha \in C_{c}(R^{N})$, *α*_*n*_ converges to $E_{C(\bar{\Omega })}\alpha$ uniformly in $R^{N}$ and thus *β*_*n*_→1 as n→∞. In addition, (3.6) together with (3.8) yields |*S*_*n*_(*w*,*Ω*)|≤*α*_*n*_(*x*) for $x\in R^{N}$ such that

3.10 $$\beta_{n}^{-1}\alpha (x)=\alpha (x)+\frac{\underset{x\in R^{N}}{\sup}|\alpha_{n}(x)-E_{C(\bar{\Omega })}\alpha (x)|}{\min_{x\in \bar{\Omega }}\alpha (x)}\alpha (x)\geq \alpha_{n}(x)\geq |S_{n}(w,\Omega )|,$$

for all *x*∈*Ω*. As a result, $\beta_{n}S_{n}(w,\Omega )\in K(D{\left(\bar{\Omega }\right)}^{d})$ and, taking account of (3.9), the proof is accomplished. ▪

**Remark 3.2 (boundary conditions)**

(i) In order to incorporate a homogeneous Dirichlet boundary condition in the context of theorem 3.1, one may use an additional reparametrization to construct a suitable approximating sequence; see [11].
(ii) If the set *K*(*W*^1,*p*^(*Ω*)^*d*^) is additionally restricted by an inhomogeneous Dirichlet boundary condition given by a function *g*∈*W*^1−1/*p*,*p*^(∂*Ω*)^*d*^ with |*g*(*x*)|≤*α*(*x*) on ∂*Ω*, the proof of theorem 3.1 fails. In fact, the sequence (3.8), which is based on the standard mollifier, does not preserve a given trace condition. In any case, the regularity of *g* (and ∂*Ω*) determines an *a priori* regularity limitation for the functions in *Y* in order to be compatible with a closure property analogous to (3.4), e.g. if $Y=C(\bar{\Omega })$ and *g*∉*C*(∂*Ω*), then *K*∩*Y* =∅. In this case, a different mollification approach needs to be pursued; cf. also §7 for an outlook on this matter.

## 4. Density results for discontinuous obstacles

### (a) Obstacles in Sobolev spaces

Note that theorem 3.1 requires continuous obstacles. In some applications, such as in the regularization and discretization of elasto-plastic contact problems or image restoration problems (see §6), it may be useful to consider obstacles that are not continuous. Under such circumstances, the following example shows that density properties of the type (3.2) or (3.4) cannot be expected if the obstacle is just a Sobolev function: without loss of generality, assume that $0\in \Omega \subset R^{N}$ with *N*≥2 and denote by

$$B_{\varepsilon }(x):=\{y\in R^{N}:|x-y{|}_{2}\leq \varepsilon \},$$

the open ball with centre $x\in R^{N}$ and radius *ε*>0 with respect to the Euclidean norm |⋅|_2_ in $R^{N}$. Let $\left\{x_{k}:k\in N\right\}$ be a countable dense subset, i.e.

$$\bar{\left\{x_{k}:k\in N\right\}}=\bar{\Omega },$$

and *r*>0 such that *B*_*r*_(0)⊂*Ω*. Consider the function

4.1 $$\varphi (x):=\tilde{\varphi }(x)\cdot \ln(\ln(c\;|x{|}_{2}^{-1})),\;c\geq \text{e}r\text{ fixed},$$

where $\tilde{\varphi }\in C_{c}^{\infty }(B_{r}(0))$ is a smooth cut-off function with $\tilde{\varphi }(x)\geq 0$ for all *x*∈*B*_*r*_(0) and $\tilde{\varphi }\equiv 1$ on *B*_*r*/2_(0). We note that *φ* is non-negative with a singularity at the origin, and its zero extension belongs to $W^{1,N}(R^{N})$; cf. [17, Example 4.43]. Further set

4.2 $$g(x):=\sum_{k=1}^{\infty }k^{-2}\varphi (x-x_{k}),\;x\in \Omega ,$$

and note that *g*∈*W*^1,*N*^(*Ω*) with *g* being unbounded at each *x*_*k*_; see [18], p.247, Example 4. Further take a function $\phi \in C^{1}(R)$ with 0≤*ϕ*(*t*)<1, *ϕ*(*t*)→1 for t→+∞ and *ϕ*′ uniformly bounded in R. By the chain rule for Sobolev functions, the obstacle

4.3 α:=2−ϕ∘g

belongs to *W*^1,*N*^(*Ω*); e.g. [14], Lemma A.3. Notice also that *α* is bounded away from zero and that it is basically equal to 1 on the dense set $\left\{x_{k}:k\in N\right\}$. Consequently, any continuous function *w* with *w*≤*α* a.e. in *Ω* fulfils *w*≤1 on *Ω*:

Assume that the latter implication is false. Then there exist $k_{0}\in N$ as well as *μ*>0,*δ*>0 such that

4.4 $$w(x)\geq 1+\mu \;\forall x\in B_{\delta }(x_{k_{0}}).$$

Let *R*>0 be such that *ϕ*(*t*)≥1−*μ*/2 for all *t*≥*R*. By continuity, there also exists *δ*′>0 such that $\varphi (x-x_{k_{0}})\geq Rk_{0}^{2}$ a.e. in *B*_*δ*′_(*x*_*k*_0__) such that

$$g(x)\geq k_{0}^{-2}\varphi (x-x_{k_{0}})\geq R,\;\text{ a.e. }x\in B_{\delta^{\prime }}(x_{k_{0}}),$$

which implies

$$w(x)\leq \alpha (x)=2-\phi (g(x))\leq 1+\frac{\mu }{2},\;\text{ a.e. }x\in B_{\delta^{\prime }}(x_{k_{0}}),$$

contradicting (4.4). Hence, any sequence of continuous functions approximating *α* from below is bounded above by 1. However, as *α*(*x*)>1 for a.e. *x*∈*Ω* by definition, and convergence in the norm topology of *L*^*p*^(*Ω*) implies convergence pointwise a.e. (along a subsequence), we obtain that

4.5 $$\alpha \in K(L^{p}(\Omega ))\setminus {\bar{K(C(\Omega )\cap L^{p}(\Omega ))}}^{L^{p}(\Omega )},$$

for any 1≤p≤+∞, and

4.6 $$\alpha \in K(W^{1,p}(\Omega ))\setminus {\bar{K(C(\Omega )\cap W^{1,p}(\Omega ))}}^{W^{1,p}(\Omega )},$$

for all *p*≤*N*, where *α* is defined by (4.3).

**Remark 4.1 (Complements on the counterexample)**

An interesting point in the preceding counterexample is the structure of the set of singularities S where *g*(*x*) is not well defined as a real number by the infinite sum (4.2), if *φ* from (4.1) is understood as a function in *C*(*Ω*∖{0}). Extending *φ* to *Ω* by setting φ(0):=+∞, we obtain $g(x_{k})=+\infty$ for all k∈N and, understanding g:Ω→R∪{+∞} as an extended real-valued function, we arrive at the following definition:

S:={x∈Ω:g(x)=+∞ with g(x) defined by (4.2) where φ(0)=+∞}.

Using the Baire category theorem, one may show that the set S is a non-meagre set with vanishing Lebesgue measure [19].

The previous construction of the counterexample is the basis for the following result.

**Theorem 4.2**

*Let* $\Omega \subset R^{N}$ *be a bounded Lipschitz domain. The following density results hold true:*

(i) *Let N≥2 and* 1≤p≤+∞*. Then there exists an obstacle* $\alpha \in W^{1,N}(\Omega )\cap L^{\infty }(\Omega )$ *satisfying (3.1) such that*
  $${\bar{K(C(\Omega )\cap L^{p}(\Omega ))}}^{L^{p}(\Omega )}⊊K(L^{p}(\Omega )),$$
  *the inclusion being strict.*
(ii) *Let N≥2 and 1≤p≤N. Then there exists an obstacle* $\alpha \in W^{1,N}(\Omega )\cap L^{\infty }(\Omega )$ *satisfying (3.1) such that
*
  $${\bar{K(C(\Omega )\cap W^{1,p}(\Omega ))}}^{W^{1,p}(\Omega )}⊊K(W^{1,p}(\Omega )),$$
  *the inclusion being strict.*
(iii) *Let* N<p<+∞ *or p=N=1. For any measurable obstacle function* α:Ω→R∪{+∞} *which satisfies (3.1), it holds that
*
  $${\bar{K(D{\left(\bar{\Omega }\right)}^{d})}}^{W^{1,p}{\left(\Omega \right)}^{d}}=K(W^{1,p}{\left(\Omega \right)}^{d}).$$

**Proof.**

We only prove assertion (iii) since (i) and (ii) follow immediately from (4.5) and (4.6). As a consequence of the Sobolev imbedding theorem, any *w*∈*K*(*W*^1,*p*^(*Ω*)^*d*^) is contained in $C{\left(\bar{\Omega }\right)}^{d}$. Let *w*∈*K*(*W*^1,*p*^(*Ω*)^*d*^). Setting

$$\hat{\alpha }(x)=\max\left(|w(x)|,\underset{x\in \Omega }{\mathrm{ess}\;\inf}\alpha (x)\right),$$

it follows that $|w(x)|\leq \hat{\alpha }(x)\text{ a.e. in }\Omega$. Since $\hat{\alpha }\in C(\bar{\Omega })$ and (3.1) holds with $\hat{\alpha }$ instead of *α*, we may invoke theorem 3.1 to infer that there exists a sequence (*w*_*n*_) with $w_{n}\in D{\left(\bar{\Omega }\right)}^{d}$, *w*_*n*_→*w* in *W*^1,*p*^(*Ω*)^*d*^ and $|w_{n}(x)|\leq \hat{\alpha }(x)\leq \alpha (x)$ a.e. in *Ω*. This entails that $w_{n}\in K(D{\left(\bar{\Omega }\right)}^{d})$ for all n∈N, which accomplishes the proof. ▪

We immediately infer the corresponding statements for Sobolev spaces incorporating homogeneous Dirichlet boundary conditions.

**Corollary 4.3**

*Let* $\Omega \subset R^{N}$ *be a bounded Lipschitz domain. The following density results hold true*:

(*i*) *Let* *N*≥2 *and* *p*≤*N*. *Then there exists an obstacle* $\alpha \in W^{1,N}(\Omega )\cap L^{\infty }(\Omega )$ *satisfying* (*3.1*) *such that*
  $${\bar{K(C(\Omega )\cap W_{0}^{1,p}(\Omega ))}}^{W_{0}^{1,p}(\Omega )}⊊K(W_{0}^{1,p}(\Omega )),$$
  *the inclusion being strict.*
(*ii*) *Let* N<p<+∞ *or* *p*=*N*=1. *For any measurable obstacle function* α:Ω→R∪{+∞} *which satisfies* (*3.1*) *it holds that*
  $${\bar{K(C_{c}^{\infty }{\left(\Omega \right)}^{d})}}^{W_{0}^{1,p}{\left(\Omega \right)}^{d}}=K(W_{0}^{1,p}{\left(\Omega \right)}^{d}).$$

**Proof.**

(i) Define the upper bound *α* by (4.3). Let $\hat{\varphi }\in C_{c}^{\infty }(\Omega )$ be a smooth cut-off function with $0\leq \hat{\varphi }\leq 1\text{ a.e. on }\Omega$ and $\hat{\varphi }\equiv 1$ except on a sufficiently small neighbourhood of ∂*Ω*. Then it holds that $\alpha \cdot \hat{\varphi }\in K(W_{0}^{1,p}(\Omega ))$ and the assertion now follows directly from the discussion preceding remark 4.1.
(ii) Taking account of (3.2), statement (ii) can be proven as theorem 4.2 (iii). ▪

### (b) Lower semicontinuous obstacles and Lebesgue spaces

The preceding counterexample provides a regularity limit in terms of the upper bound *α* for which the density property (3.2) in the space *X*(*Ω*)=*L*^*p*^(*Ω*)^*d*^ can be expected to hold. In this regard, however, uniform continuity is far from being a necessary condition. In order to enlarge the space of obstacles compatible with (3.2), we first consider upper bounds that allow for a lower semicontinuous representative, i.e. there exists a lower semicontinuous function in the equivalence class of functions that are Lebesgue-almost everywhere equal to *α*.

**Theorem 4.4**

*Let* $\Omega \subset R^{N}$ *be a bounded Lipschitz domain and* 1≤p<+∞*. If* α:Ω→R∪{+∞} *has a lower semicontinuous representative that fulfils (3.1), then it holds that
*

$${\bar{K(C_{c}^{\infty }{\left(\Omega \right)}^{d})}}^{L^{p}{\left(\Omega \right)}^{d}}=K(L^{p}{\left(\Omega \right)}^{d}).$$

**Proof.**

Let *w*∈*K*(*L*^*p*^(*Ω*)^*d*^). Consider a lower semicontinuous function α:Ω→R∪{+∞} that fulfils (3.1). Without loss of generality, we may assume that $\underset{x\in \Omega }{\inf}\alpha (x)>0$. Denote by $\tilde{\alpha }$ the extension of *α* given by $\tilde{\alpha }(x):=\alpha (x),x\in \Omega$, $\tilde{\alpha }(x):=\underset{x\in \Omega }{\inf}\alpha (x)$ on $R^{N}\setminus \Omega$, and note that $\tilde{\alpha }$ is lower semicontinuous (l.s.c.) on $R^{N}$. The Lipschitz regularization of $\tilde{\alpha }$,

$$\alpha_{n}(x)=\underset{y\in R^{N}}{\inf}\{\tilde{\alpha }(y)+n∥x-y∥\},$$

yields a sequence (*α*_*n*_) with $\alpha_{n}\in C(\bar{\Omega })$, $\underset{x\in \Omega }{\inf}\alpha (x)\leq \alpha_{n}(x)\leq \alpha (x)$ for all x∈Ω,n∈N and *α*_*n*_(*x*)→*α*(*x*) a.e. in *Ω*; see, e.g. [2], Theorem 9.2.1. Now consider the functions

$$w_{n}(x):=\min\{|w(x)|,\alpha_{n}(x)\}\frac{w(x)}{|w(x)|},$$

where it is understood that *w*_*n*_(*x*):=0 if *w*(*x*)=0. It follows from Lebesgue's theorem on dominated convergence that *w*_*n*_→*w* in *L*^*p*^(*Ω*)^*d*^. Further observe that *w*_*n*_∈*K*_*n*_(*L*^*p*^(*Ω*)^*d*^) where

$$K_{n}(X(\Omega )):=\{w\in X(\Omega ):|w(x)|\leq \alpha_{n}(x)\text{ a.e. }\text{on }\Omega \}.$$

Let *ε*>0. According to (3.2), for each n∈N, *w*_*n*_ can be approximated by a smooth function ${\tilde{w}}_{n}\in K_{n}(C_{c}^{\infty }{\left(\Omega \right)}^{d})\subset K(C_{c}^{\infty }{\left(\Omega \right)}^{d})$ such that

$$∥w_{n}-{\tilde{w}}_{n}{∥}_{L^{p}{\left(\Omega \right)}^{d}}<\frac{\varepsilon }{2}.$$

For sufficiently large *n*, we conclude that

4.7 $$∥w-{\tilde{w}}_{n}{∥}_{L^{p}{\left(\Omega \right)}^{d}}\leq ∥w-w_{n}{∥}_{L^{p}{\left(\Omega \right)}^{d}}+∥w_{n}-{\tilde{w}}_{n}{∥}_{L^{p}{\left(\Omega \right)}^{d}}<\frac{\varepsilon }{2}+\frac{\varepsilon }{2}=\varepsilon ,$$

which concludes the proof. ▪

We proceed by considering the important special case of a piecewise continuous upper bound; suppose there exists a partition of *Ω* into open subsets *Ω*_*l*_⊂*Ω* with Lipschitz boundary such that $\bar{\Omega }=\cup_{l=1}^{L}{\bar{\Omega }}_{l}$, *Ω*_*i*_∩*Ω*_*j*_=∅ for *i*≠*j* and

4.8 $$\alpha {|}_{\Omega_{l}}\in C({\bar{\Omega }}_{l}),\;\underset{x\in \Omega_{l}}{\inf}\alpha {|}_{\Omega_{l}}(x)>0,\;l=1,\ldots ,L.$$

Theorem 4.4 ensures that for obstacles of this class the density result in the norm topology of the *L*^*p*^-spaces holds true.

### (c) Lower semicontinuous obstacles and Sobolev spaces

Conditions on the obstacle *α* so that the density results for Sobolev spaces hold can be relaxed from assuming that $\alpha \in C(\bar{\Omega })$ to lower regularity requirements with the aid of Mosco convergence of closed and convex sets. The following definition goes back to [15].

**Definition 4.5 (Mosco convergence)**

Let *X* be a reflexive Banach space and (*K*_*n*_) a sequence of closed convex subsets with *K*_*n*_⊂*X* for all n∈N. Then *K*_*n*_→*MK* as n→+∞, i.e. (*K*_*n*_) is said to Mosco converge to the set *K*⊂*X*, if and only if

M 1  K ⊃{v∈X:( ∃ (vk)⊂X:vk∈Knk∀k∈N,vk⇀v)}

M 2  andK ⊂{v∈X:( ∃ (vn)⊂X,  ∃ N∈N:vn∈Kn ∀n≥N,vn→v)}.

Here, (*K*_*n*_*k*__) denotes an arbitrary subsequence of (*K*_*n*_) and the subset notation (*v*_*k*_)⊂*X* has to be understood in the sense that {*v*_*k*_}⊂*X*. The following class of obstacles encompasses functions in *W*^1,*q*^(*Ω*) that fulfil a generalized lower semicontinuity condition.

**Definition 4.6**

We denote by $W^{q}(\Omega )$ for *q*≥1 the set of functions *α*∈*W*^1,*q*^(*Ω*) for which there exists a sequence of functions (*α*_*n*_) with *α*_*n*_ satisfying (3.1), *α*_*n*_≤*α* a.e. in *Ω* and $\alpha_{n}\in C(\bar{\Omega })\cap W^{1,q}(\Omega )$ for all n∈N such that $\alpha_{n}⇀\alpha$ in *W*^1,*q*^(*Ω*).

Note that the class $W^{q}(\Omega )$ is strictly contained in *W*^1,*q*^(*Ω*). Additionally, any obstacle $\alpha \in W^{q}(\Omega )$ has a lower semicontinuous representative, which follows easily from definition 4.6 and by extraction of a pointwise almost everywhere converging subsequence. However, the functions in $W^{q}$ are not necessarily continuous: it suffices to consider the example from (4.1) for *Ω*=*B*_*r*_(0), *N*>1 and

4.9 $$\alpha (x)=\ln(\ln(c|x{|}^{-1})),\;c\geq \text{e}r\text{ fixed}.$$

It follows that *α*∈*W*^1,*q*^(*Ω*) for all *q*≤*N* (see [17], Example 4.43), $\alpha \notin C(\bar{\Omega })$, and the sequence (*α*_*n*_) defined as $\alpha_{n}(x)=\min(\alpha (x),n)$ for n∈N satisfies the requirements of the definition of $W^{q}(\Omega )$.

**Theorem 4.7**

*Let* $\Omega \subset R^{N}$ *be a bounded Lipschitz domain. Let* 1≤p<∞ *and* $\alpha \in W^{p}(\Omega )$*. Then the following density results hold true:
*

$$\begin{array}{rl} {\bar{K(D{\left(\Omega \right)}^{d};|\cdot {|}_{\infty })}}^{W_{0}^{1,p}{\left(\Omega \right)}^{d}} & \;=K(W_{0}^{1,p}{\left(\Omega \right)}^{d};|\cdot {|}_{\infty })\\ \mathrm{and}\;\;{\bar{K(D{\left(\bar{\Omega }\right)}^{d};|\cdot {|}_{\infty })}}^{W^{1,p}{\left(\Omega \right)}^{d}} & \;=K(W^{1,p}{\left(\Omega \right)}^{d};|\cdot {|}_{\infty }), \end{array}$$

*where* $K(X(\Omega );|\cdot {|}_{\infty })=\{w\in X(\Omega ):|w(x){|}_{\infty }\leq \alpha (x)\text{ a.e. }x\in \Omega \}$.

**Proof.**

Without loss of generality, consider the one-dimensional case *d*=1. Let $w\in K(W_{0}^{1,p}(\Omega );|\cdot {|}_{\infty })$ and (*α*_*n*_)⊂*W*^1,*p*^(*Ω*) according to definition 4.6. By Mazur's lemma, we may as well assume that (*α*_*n*_) converges strongly to *α* in *W*^1,*p*^(*Ω*) since convex combinations preserve order and continuity. Hence, one obtains the Mosco convergence result

$$K_{n}^{\pm }(W_{0}^{1,p}(\Omega ))\overset{M}{⟶}K^{\pm }(W_{0}^{1,p}(\Omega ))$$

for the unilateral constraint sets

$$\begin{array}{rl} K_{n}^{-}(X(\Omega )) & \;:=\{w\in X(\Omega ):w(x)\geq -\alpha_{n}\text{ a.e. in }\Omega \},\\ K_{n}^{+}(X(\Omega )) & \;:=\{w\in X(\Omega ):w(x)\leq \alpha_{n}\text{ a.e. in }\Omega \},\\ K_{-}(X(\Omega )) & \;:=\{w\in X(\Omega ):w(x)\geq -\alpha \text{ a.e. in }\Omega \}\\ \mathrm{and}\;\;K_{+}(X(\Omega )) & \;:=\{w\in X(\Omega ):w(x)\leq \alpha \text{ a.e. in }\Omega \}. \end{array}$$

Consequently, there exist two recovery sequences,

4.10 $$w_{n}^{\pm }\in K_{n}^{\pm }(W_{0}^{1,p}(\Omega )),$$

with $w_{n}^{\pm }\to w$ in $W_{0}^{1,p}(\Omega )$. Using the continuity of

$$\max(\ldots ,0),\min(\ldots ,0):\;W_{0}^{1,p}(\Omega )\to W_{0}^{1,p}(\Omega ),$$

it follows that the sequence

$$w_{n}=\max(w_{n}^{+},0)+\min(w_{n}^{-},0),$$

converges to *w* in $W_{0}^{1,p}(\Omega )$. Moreover, it holds that |*w*_*n*_|≤*α*_*n*_ for all n∈N. For each n∈N, the assumptions on *α*_*n*_ allow to use (3.2) to infer the existence of a smooth function ${\tilde{w}}_{n}\in C_{c}^{\infty }(\Omega )$ with $|{\tilde{w}}_{n}|\leq \alpha_{n}\leq \alpha \text{ a.e. in }\Omega$ that approximates *w*_*n*_ arbitrarily well. Using *w*_*n*_→*w* in $W_{0}^{1,p}{\left(\Omega \right)}^{d}$, the assertion follows by an *ε*/2-argument as in (4.7). The proof for the case *X*(*Ω*)=*W*^1,*p*^(*Ω*)^*d*^ follows analogously by invoking theorem 3.1. ▪

### (d) Supersolutions of elliptic partial differential equations

By now, density properties for pointwise constraints in Sobolev spaces of the type

$${\bar{K(C_{c}^{\infty }{\left(\Omega \right)}^{d})}}^{W_{0}^{1,p}{\left(\Omega \right)}^{d}}=K(W_{0}^{1,p}{\left(\Omega \right)}^{d}),\;\text{or}\;{\bar{K(D{\left(\bar{\Omega }\right)}^{d})}}^{W^{1,p}{\left(\Omega \right)}^{d}}=K(W^{1,p}{\left(\Omega \right)}^{d}),$$

have been obtained on the basis of mollification and a subsequent procedure to enforce feasibility. An alternative approach is the approximation of a function via the solution of an appropriate sequence of elliptic PDEs. Using standard regularity theory, one may prove higher regularity of the approximating sequence and one is left to prove feasibility. In this section, we focus on obstacles which are solutions of an elliptic PDE. Therefore, consider a general second-order differential operator *A* in divergence form;

4.11 $$A=\sum_{i,j=1}^{N}-\frac{\partial }{\partial x_{i}}a_{ij}(x)\frac{\partial }{\partial x_{j}}+\sum_{i=1}^{n}b_{i}(x)\frac{\partial }{\partial x_{i}}+c(x),$$

where $a_{ij},b_{i},c\in L^{\infty }(\Omega )$ for 1≤*i*,*j*≤*N*. Here, the matrix [*a*_*ij*_(*x*)] is symmetric a.e. and uniformly elliptic, i.e. there exists a *κ*_*a*_>0 such that

$$\sum_{i,j=1}^{N}a_{ij}(x)\xi_{i}\xi_{j}\geq \kappa_{a}|\xi {|}^{2},\;\forall \xi \in R^{N},$$

for a.e. *x*∈*Ω*. It is further assumed that *a*_*ij*_,*b*_*i*_,*c* are such that $A:H_{0}^{1}(\Omega )\to H^{-1}(\Omega )$ is strongly monotone, i.e. there exists *κ*>0 such that

$$\langle Au,u\rangle \geq \kappa ∥u{∥}_{H_{0}^{1}(\Omega )}^{2},\;\forall u\in H_{0}^{1}(\Omega ),$$

where 〈…,…〉 denotes the duality pairing in *H*^−1^(*Ω*). For example, this is the case if *b*_*i*_≡0 for 1≤*i*≤*N* and *c*(*x*)≥0 a.e. in *Ω*. We call a function *α*∈*H*^1^(*Ω*) weak supersolution with respect to the elliptic operator *A*, if *Aα*≥0 in the *H*^−1^(*Ω*)-sense, that is

4.12 $$\langle A\alpha ,v\rangle \geq 0,\;\forall v\in H_{0}^{1}(\Omega ),v\geq 0\text{ a.e. in }\Omega .$$

The subsequent theorem covers density properties for obstacles that are weak supersolutions of an elliptic PDE of type (4.11).

**Theorem 4.8**

*Let Ω be a bounded domain. Let α∈H*^1^*(Ω) be a weak supersolution for some A as in (4.11) in the sense of (4.12), with α≥0 on ∂Ω. For* $X(\Omega )\in \{L^{2}{\left(\Omega \right)}^{d},H_{0}^{1}{\left(\Omega \right)}^{d}\}$*, it holds that
*

$${\bar{K(Y(\Omega ),|\cdot {|}_{\infty })}}^{X(\Omega )}=K(X(\Omega ),|\cdot {|}_{\infty }),$$

*in the following cases:*

(i) $a_{ij}\in C^{0,1}(\bar{\Omega })$ *or a*_*ij*_*∈C*^1^*(Ω):* $Y(\Omega )={\left(H_{\mathrm{loc}}^{2}(\Omega )\cap H_{0}^{1}(\Omega )\right)}^{d}$*,*
(ii) *∂Ω∈C*^1,1^ *or Ω convex,* $a_{ij}\in C^{0,1}(\bar{\Omega })$*:* $Y(\Omega )={\left(H^{2}(\Omega )\cap H_{0}^{1}(\Omega )\right)}^{d}$*,*
(iii) *a*_*ij*_*,b*_*i*_*,c∈C*^*m*+1^*(Ω),* $m\in N_{0}$*:* $Y(\Omega )={\left(H_{\mathrm{loc}}^{m+2}(\Omega )\cap H_{0}^{1}(\Omega )\right)}^{d}$ and
(iv) *∂Ω∈C*^*m*+2^*,* $a_{ij},b_{i},c\in C^{m+1}(\bar{\Omega })$*,* $m\in N_{0}$*:* $Y(\Omega )={\left(H^{m+2}(\Omega )\cap H_{0}^{1}(\Omega )\right)}^{d}$.

**Proof.**

Without loss of generality, assume *d*=1. First observe that the maximum principle implies *α*(*x*)≥0 a.e. in *Ω*. Let *w*∈*K*(*X*(*Ω*)) be arbitrary. Consider the sequence (*w*_*n*_), where *w*_*n*_ is defined as the unique solution to the problem,

4.13 $$\text{find }y\in H_{0}^{1}(\Omega ):\;\frac{1}{n}Ay+y=w\;\text{ in }H^{-1}(\Omega ).$$

We denote by *T*_*n*_ the solution mapping to (4.13), i.e. *w*_*n*_=*T*_*n*_(*w*).

*Step 1*: *T*_*n*_-invariance of $K(H_{0}^{1}(\Omega ))$: We now prove that for any n∈N, we have that −*α*≤*w*_*n*_≤*α* a.e., i.e.

4.14 $$T_{n}:K(L^{2}(\Omega ))\to K(H_{0}^{1}(\Omega )),$$

given that *Aα*≥0 in the *H*^−1^(*Ω*). Proceeding as in [20], we consider (*w*_*n*_−*α*)^+^ as a test function on (4.13) and add to both sides −〈(1/*n*)*Aα*+*α*,(*w*_*n*_−*α*)^+^〉. Then,

$$\begin{array}{rl} \frac{\kappa }{n}∥{\left(w_{n}-\alpha \right)}^{+}{∥}_{H_{0}^{1}(\Omega )}^{2}+∥{\left(w_{n}-\alpha \right)}^{+}{∥}_{L^{2}(\Omega )}^{2} & \;\leq \left\langle \left(\frac{1}{n}A+I\right)(w_{n}-\alpha ),{\left(w_{n}-\alpha \right)}^{+}\right\rangle \\ & \;\leq \left\langle w-\alpha -\frac{1}{n}A\alpha ,{\left(w_{n}-\alpha \right)}^{+}\right\rangle \\ & \;\leq -\frac{1}{n}\langle A\alpha ,{\left(w_{n}-\alpha \right)}^{+}\rangle \leq 0, \end{array}$$

where we have used that *w*−*α*≤0 a.e. in *Ω*. Therefore, *w*_*n*_≤*α* a.e. in *Ω*. Analogously, we obtain that *w*_*n*_≥−*α* a.e. by considering (−*α*−*w*_*n*_)^+^ as a test function and by adding to both sides −〈(1/*n*)*Aα*+*α*,(−*α*−*w*_*n*_)^+^〉. This proves (4.14), i.e. $w_{n}\in K(H_{0}^{1}(\Omega ))$.

*Step 2*: Some convergence results for singular perturbations.

The desired convergence modes of the approximating sequences rely on standard arguments for singular perturbations, cf. [7], Theorems 9.1 and 9.4 for the case of singularly perturbed variational inequalities. First, for *y*∈*L*^2^(*Ω*) it holds

4.15 $$\lim_{n\to \infty }y_{n}=y\text{ in }L^{2}(\Omega )\;⟹\;{\hat{y}}_{n}:=T_{n}(y_{n})\to y\text{ in }L^{2}(\Omega ).$$

Second, for $y\in H_{0}^{1}(\Omega )$, we prove that

4.16 $$\lim_{n\to \infty }y_{n}=y\text{ in }H_{0}^{1}(\Omega )\;⟹\;\lim_{n\to \infty }{\hat{y}}_{n}=y\text{ in }H_{0}^{1}(\Omega ).$$

In fact, since $y_{n}\in H_{0}^{1}(\Omega )$ and *A* is strongly monotone, we observe that

$$\begin{array}{rl} \frac{\kappa }{n}∥{\hat{y}}_{n}-y_{n}{∥}_{H_{0}^{1}(\Omega )}^{2}+∥{\hat{y}}_{n}-y_{n}{∥}_{L^{2}(\Omega )}^{2} & \;\leq \left\langle \left(\frac{1}{n}A+I\right)({\hat{y}}_{n}-y_{n}),{\hat{y}}_{n}-y_{n}\right\rangle \\ & \;=\frac{1}{n}\langle Ay_{n},y_{n}-{\hat{y}}_{n}\rangle \\ & \;\leq \frac{1}{n}∥Ay_{n}{∥}_{H^{-1}(\Omega )}∥y_{n}-{\hat{y}}_{n}{∥}_{H_{0}^{1}(\Omega )}, \end{array}$$

where we have used that ${\hat{y}}_{n}$ solves (4.13) with *y*_*n*_ as right-hand side. Hence $\left({\hat{y}}_{n}\right)$ is bounded in $H_{0}^{1}(\Omega )$. Employing (4.15) one obtains that ${\hat{y}}_{n}⇀y$ in $H_{0}^{1}(\Omega )$ along a subsequence, and by uniqueness, it holds ${\hat{y}}_{n}⇀y$ for the entire sequence $\left({\hat{y}}_{n}\right)$. Finally, from the inequalities above, we have

$$\kappa \underset{n\to \infty }{lim sup}|{\hat{y}}_{n}-y_{n}{|}_{H_{0}^{1}(\Omega )}^{2}\leq \underset{n\to \infty }{lim sup}\langle Ay_{n},y_{n}-{\hat{y}}_{n}\rangle =0,$$

so that ${\hat{y}}_{n}=T_{n}(y_{n})\to y$ in $H_{0}^{1}(\Omega )$ and thus (4.16) is proven.

Thirdly, in addition to *w*_*n*_=*T*_*n*_(*w*), we define $w_{n}^{q}=T_{n}^{q}(w)$ where $T_{n}^{q}(w):=T_{n}(T_{n}^{q-1}(w))$ for q∈N,q≥2, $T_{n}^{1}(w):=T_{n}(w)=w_{n}$ and $w_{n}^{0}:=w$. It can be deduced from (4.15) and (4.16) by induction that

4.17 $$\lim_{n\to \infty }w_{n}^{q}=w\;\text{in }L^{2}(\Omega ),\;\forall q\in N\cup \{0\},$$

for *w*∈*L*^2^(*Ω*), and

4.18 $$\lim_{n\to \infty }w_{n}^{q}=w\;\text{in }H_{0}^{1}(\Omega ),\;\forall q\in N\cup \{0\},$$

for $w\in H_{0}^{1}(\Omega )$, respectively.

*Step 3*: Regularity and convergence of the approximating sequences

The extra regularity of the $H_{0}^{1}(\Omega )$-solution *T*_*n*_(*w*) to (4.13) is different with respect to the statement cases: if $a_{ij}\in C^{0,1}(\bar{\Omega })$ or *a*_*ij*_∈*C*^1^(*Ω*) for 1≤*i*,*j*≤*N*, the solution *T*_*n*_(*w*) belongs to $H_{0}^{1}(\Omega )\cap H_{\mathrm{loc}}^{2}(\Omega )$ (see [21] for the first case and [18] for the second one). The solution *T*_*n*_(*w*) belongs to $H_{0}^{1}(\Omega )\cap H^{2}(\Omega )$ if ∂*Ω* is *C*^1,1^-smooth [21] or when *Ω* is convex [22].

In case *w*∈*K*(*L*^2^(*Ω*)), (4.15) with *y*_*n*_≡*w* ensures that *w*_*n*_→*w* in *L*^2^(*Ω*). In conjunction with the regularity and the feasibility of *w*_*n*_=*T*_*n*_(*w*) described above, we have then established (i) and (ii) for *X*(*Ω*)=*L*^2^(*Ω*). Secondly, note that if $w\in K(H_{0}^{1}(\Omega ))$, then *w*_*n*_→*w* in $H_{0}^{1}(\Omega )$ by (4.16) with *y*_*n*_≡*w*, and as seen above, $w_{n}\in K(H_{0}^{1}(\Omega ))$. This, together with the regularity of *w*_*n*_=*T*_*n*_(*w*) established above, proves in turn (i) and (ii) for $X(\Omega )=H_{0}^{1}(\Omega )$.

It is left to argue for (iii) and (iv) as follows. If *a*_*ij*_,*b*_*i*_,*c*∈*C*^*m*+1^(*Ω*) for 1≤*i*,*j*≤*N*, then for each n∈N, the operator *T*_*n*_ has the following increasing regularity properties [18],

$$w\in H_{\mathrm{loc}}^{k}(\Omega )⟹T_{n}(w)\in H_{\mathrm{loc}}^{k+2}(\Omega )\cap H_{0}^{1}(\Omega ),\;0\leq k\leq m;$$

and if $a_{ij},b_{i},c\in C^{m+1}(\bar{\Omega })$ for 1≤*i*,*j*≤*N* and ∂*Ω* is of class *C*^*m*+2^, for each n∈N,

$$w\in H^{k}(\Omega )⟹T_{n}(w)\in H^{k+2}(\Omega )\cap H_{0}^{1}(\Omega ),\;0\leq k\leq m.$$

Finally, this proves (iii) given that $w_{n}^{q}\in H_{\mathrm{loc}}^{m+2}(\Omega )\cap H_{0}^{1}(\Omega )$ for 2*q*≥*m*+2, $w_{n}^{q}\in K(H_{0}^{1}(\Omega ))$, and $w_{n}^{q}\to w$ as n→∞ in *L*^2^(*Ω*) or $H_{0}^{1}(\Omega )$ depending on the regularity of *w*, cf. (4.17) and (4.18). The analogous reasoning applies to (iv). ▪

Let us briefly comment on the relation to the density results from theorem 4.4 and theorem 4.7. First, note that we do not require the obstacle to be bounded away from zero as we did in the preceding paragraphs. Secondly, the maximal regularity of the feasible approximation hinges on the coefficients of the elliptic operator associated with the obstacle and the smoothness of the boundary. Concerning the semicontinuity requirements of the upper bound, a classical result from Trudinger [23], Cor. 5.3 for the case without lower order terms (*b*_*i*_≡0,*c*≡0) states that any weak supersolution in the sense of (4.12) is upper semicontinuous. By contrast, the consideration of upper bounds that are weak subsolutions of an elliptic PDE is not useful as these functions may easily fail to be non-negative on *Ω*. For example, this is the case if a weak subsolution satisfies a Dirichlet boundary condition.

## 5. Application to finite elements

### (a) Finite-element discretized convex sets

In the following, we investigate the issue of the Mosco convergence (definition 4.5) of finite-dimensional approximations *K*_*n*_ of a convex constraint set *K*(*X*(*Ω*)) of the type (3.3); see §2b(ii) for a general motivation in the context of variational inequality problems. In this section, it is assumed that the sets (*K*_*n*_) result from a suitable finite-element discretization such that the parameter *n* is associated with a sequence of mesh widths (*h*_*n*_) tending to zero. The convergence of (*K*_*n*_) in the sense of definition 4.5 ensures that the solutions of the discrete problems converge to the solution of the original infinite-dimensional problem irrespectively of the regularity of the data or the obstacle defining *K*(*X*(*Ω*)); see [7], ch. 4, Theorem 4.1. Mosco convergence results of this type are rarely found in the literature and are typically confined to simpler constraint sets and higher regularity assumptions on the obstacle; see, for instance, [4] for the case of an $H^{1}(\Omega )\cap C(\bar{\Omega })$-bound in the context of the obstacle problem. The density results from the preceding sections provide the basis for new Mosco convergence results under minimal regularity (of the solution) and under weaker assumptions on the regularity of the obstacle *α*. We further provide novel Mosco convergence results for discretized constraints on partial derivatives, including Raviart–Thomas finite-element approaches for problems in *H*(div). As a general rule, density results of the type (1.1) represent a powerful means to verify the convergence of finite-element methods for convex constrained problems under minimal regularity. Applications involving constraint sets of the type (3.3) with low regularity of *α* are manifold and comprise, for instance, the discretization of variational problems in mechanics, such as in elasto-plasticity with hardening [24], and in image restoration, with regard to the predual problem of TV-regularization [25]. Moreover, the issue occurs in fixed point-based approaches to the solution of quasi-variational inequalities through the implicit definition of obstacles.

**Remark 5.1**

In some textbooks on finite-dimensional approximations of variational inequalities, cf. e.g. [4,6], condition (M2) is replaced by the following criterion:

M2′ $$\begin{array}{cc} \; & \begin{array}{rl} & \text{there exists a dense subset }\tilde{K}\subset K\text{ and an operator }r_{n}:\tilde{K}\to X\\ & \text{such that for all }v\in \tilde{K}\text{ it holds }r_{n}v\to v\text{ in }X\text{ and there exists }n_{0}\in N\\ & \text{such that }r_{n}v\in K_{n}\text{ for all }n\geq n_{0}. \end{array}\} \end{array}$$

It is easy to show that (M2′) implies (M2). In fact, let *v*∈*K* and denote by *π*_*K*_*n*__*v* its (not necessarily uniquely determined) projection onto *K*_*n*_. By density, for *ε*>0, there exists $v^{\varepsilon }\in \tilde{K}$ such that ∥*v*^*ε*^−*v*∥≤*ε*. Thus, it holds

$$∥v-\pi_{K_{n}}v∥=\underset{v^{n}\in K_{n}}{\inf}∥v-v^{n}∥\leq ∥v-r_{n}v^{\varepsilon }∥\leq \varepsilon +∥v^{\varepsilon }-r_{n}v^{\varepsilon }∥,$$

for sufficiently large *n* such that $\lim_{n\to \infty }∥v-\pi_{K_{n}}v∥\leq \varepsilon$, where *ε* was arbitrary.

The condition (M2′) turns out to be convenient especially in the context of finite-dimensional approximations, where (*r*_*n*_) is given by suitable interpolation operators, which typically are only well defined on a dense subset *Y* (*Ω*) of *X*(*Ω*) giving rise to sets $\tilde{K}$ of the type *K*(*Y* (*Ω*)). This is precisely the point where the density results of §3 are needed.

Note that Mosco convergence is a powerful tool whenever the discrete spaces are fixed *a priori*, i.e. regardless of the data of the specific problem. The resulting sequence of finite-dimensional problems can be understood as an approximation of *any problem in a given problem class*.

By contrast, *adaptive* finite-element methods intend to design the sets *K*_*n*_ in order to approximate *the solution of a specific problem*. However, rigorous convergence proofs with regard to adaptive discretizations of variational inequalities are restricted to special cases and usually rely on rather strong assumptions. For instance, in the case of the obstacle problem with a piecewise affine obstacle, we mention the article [26]. Moreover, density results may still be useful in the convergence analysis of adaptive schemes which require interpolation operators (cf. [27]).

### (b) Finite-element spaces and interpolation operators

In this section, we assume that $\Omega \subset R^{N}$ is polyhedral. Together with *Ω*, a sequence of geometrically conforming affine simplicial meshes ${\left(T_{h}\right)}_{h>0}$ of *Ω* with mesh size

$$h:=\max_{T\in T_{h}}\mathrm{diam}T$$

is assumed to be given. For details, we refer to [28]. In analogy to the case *N*=2, we refer to each $T_{h}$ as a triangulation. The (*N*-dimensional) Lebesgue measure of an element $T\in T_{h}$ is denoted by *λ*(*T*). We also admit the standard assumption that the sequence $\left(T_{h}\right)$ is shape-regular, i.e.

5.1 $$\;\exists \;c>0:\;\frac{\mathrm{diam}(T)}{\rho_{T}}\leq c\;\forall h\;\forall T\in T_{h},$$

where $\mathrm{diam}(T)=\max_{x,y\in T}|x-y|$ denotes the diameter of *T* and *ρ*_*T*_ designates the diameter of the largest ball that is contained in *T*. We further write *x*_*T*_ for the (barycentric) midpoint of an element *T*, and $M_{h}=\{x_{T}:T\in T_{h}\}$, $N_{h}$ and $E_{h}$ for the set of element midpoints, triangulation nodes and edges with respect to $T_{h}$, respectively. By abuse of notation, we write $|M_{h}|$ and $|N_{h}|$ for the cardinality of the respective set. Let $\chi_{T}:\Omega \to R$ designate the characteristic function of *T* with respect to *Ω*, that is

$$\chi_{T}(x)=0,\;\forall x\notin T,\;\chi_{T}(x)=1,\;\forall x\in T.$$

We further make use of the standard *H*^1^(*Ω*)-conforming finite-element space of globally continuous, piecewise affine functions denoted by

$$P_{1,h}(\Omega ):=\{u\in C(\bar{\Omega }):u{|}_{T}\in P_{1}\;\forall T\in T_{h}\}.$$

Here, $P_{1}$ denotes the space of polynomials of degree less than or equal to one. Together with the finite-dimensional subspace *P*_1,*h*_(*Ω*) and its standard nodal basis $\left\{\varphi_{x}:x\in N_{h}\right\}$, we consider the global interpolation operator

5.2 $$I_{h}:C(\bar{\Omega })\to P_{1,h}(\Omega )\;\mathrm{and}\;I_{h}u:=\sum_{x\in N_{h}}u(x)\varphi_{x}.$$

Note that *I*_*h*_ is only defined on a dense subspace of *H*^1^(*Ω*). For the discretization of variational problems in *H*(div;*Ω*), it is customary to use the conforming space of Raviart–Thomas finite elements of lowest order

5.3 $${\mathrm{RT}}_{h}(\Omega ):=\{w\in L^{2}{\left(\Omega \right)}^{N}:w{|}_{T}\in RT\;\forall T\in T_{h},[w\cdot \nu ]{|}_{E\cap \Omega }=0\;\forall E\in E_{h}\},$$

where $RT:=\{w\in P_{1}^{d}:\;\exists \;a\in R^{d},b\in R:w(x)=a+bx\}$ and *ν* denotes the unit outer normal to *T*. To incorporate homogeneous Neumann boundary conditions, one uses the *H*_0_(div;*Ω*)-conforming subspace

$$RT_{0,h}(\Omega ):=RT_{h}(\Omega )\cap H_{0}(\mathrm{div};\Omega ).$$

The construction of suitable edge-based basis functions $\left\{\varphi_{E}:E\in E_{h}\right\}$ can be found in the literature, cf., for instance, [29], such that the boundary condition in the definition of *RT*_0,*h*_(*Ω*) can be easily accounted for. The global Raviart–Thomas interpolation operator is given by

5.4 $$I_{h}^{\mathrm{RT}}:W^{1,1}{\left(\Omega \right)}^{N}\to {\mathrm{RT}}_{h}(\Omega ),\;I_{h}^{\mathrm{RT}}w:=\sum_{E\in E_{h}}\left(\int_{E}w\cdot \nu \;dH^{N-1}\right)\varphi_{E}.$$

### (c) Mosco convergence results under minimal regularity

We emphasize that the subsequent results may be extended to finite elements of higher order, which are typically useful when the solution to the variational problem, e.g. (2.8), displays a higher regularity. In this regard, higher regularity assumptions on the data and the obstacle are required and the concept of Mosco convergence is not binding to prove the convergence of the finite-element method, and *a priori* error estimates with a rate can be derived (cf. e.g. [30]). However, we do not want to deviate from minimal regularity assumptions on the data. Further, even for simple variational problems such as the classical elasto-plastic torsion problem, there is a regularity limitation for the solution regardless of the smoothness of the data (cf. [4]).

Note also that the subsequently covered problems comprise situations where the discrete feasible sets *K*_*h*_ are not necessarily nested and non-conforming in the sense that they are in general not contained in the feasible set *K*(*X*). In the following, *c* denotes a positive constant, which may take different values on different occasions.

**Lemma 5.2**

*Let* $\Omega \subset R^{N}$ *be a polyhedral domain and* $\alpha \in C(\bar{\Omega })$ *with* *α*(*x*)≥0 *in* *Ω*. *Further let* (*w*_*h*_) *be a sequence that fulfils for all* *h*, *w*_*h*_∈*P*_1,*h*_(*Ω*)^*d*^ *and* |*w*_*h*_(*x*_*T*_)|≤*α*(*x*_*T*_) *for all* $T\in T_{h}$. *If* $w_{h}⇀w$ *for* *h*→0 *in* *L*^2^(*Ω*)^*d*^, *then it holds that* |*w*|≤*α* *a.e. in* *Ω*.

**Proof.**

It suffices to show that *i*_*K*_(*w*)=0, where

$$K:=\{w\in L^{2}{\left(\Omega \right)}^{d}:|w|\leq \alpha \text{ a.e.}\}.$$

Moreover, it holds that *i*_*K*_=j*, where j* denotes the Fenchel conjugate

$$j^{*}(v^{*}):=\underset{v\in L^{2}{\left(\Omega \right)}^{d}}{\sup}\{(v^{*},v)-j(v)\},$$

of the mapping $j:L^{2}{\left(\Omega \right)}^{d}\to R,\;j(v):=\int_{\Omega }\alpha |v{|}_{*}\;dx$. Here,

$$|v^{*}{|}_{*}=\underset{v\in R^{d}\setminus \{0\}}{\sup}v^{*}\cdot v/|v|$$

denotes the dual norm of |⋅|. From the definition of j*, we obtain that *i*_*K*_(*w*)=0 is equivalent to

5.5 $$(w,v)\leq \int_{\Omega }\alpha |v{|}_{*}\;\forall v\in L^{2}{\left(\Omega \right)}^{d}.$$

By a density argument, it suffices to prove this result for all *v*∈*C*_*c*_(*Ω*)^*d*^. Denote by

5.6 $$\alpha_{h}:=\sum_{T\in T_{h}}\alpha (x_{T})\chi_{T}\;\mathrm{and}\;v_{h}:=\sum_{T\in T_{h}}v(x_{T})\chi_{T},$$

the piecewise constant interpolants of *α* and *v*, respectively. By definition of (*a*_*h*_) and (*v*_*h*_) as well as the uniform continuity of *α* and *v* it follows that *α*_*h*_→*α* and *v*_*h*_→*v*, both in $L^{\infty }(\Omega )$. By the weak convergence of (*w*_*h*_), the strong convergence of (*α*_*h*_) and (*v*_*h*_) as well as the midpoint quadrature rule, we obtain

5.7 $$\begin{array}{rl} \int_{\Omega }w\cdot v\;dx\leftarrow \int_{\Omega }w_{h}\cdot v_{h}\;dx & \;=\sum_{T\in T_{h}}\int_{T}w_{h}\cdot v_{h}\;dx\\ & \;=\sum_{T\in T_{h}}\lambda (T)\;w_{h}(x_{T})\cdot v_{h}{|}_{T}\;dx\\ & \;\leq \sum_{T\in T_{h}}\lambda (T)\alpha (x_{T})|v_{h}{|}_{T}{|}_{*}\;dx\\ & \;=\int_{\Omega }\alpha_{h}|v_{h}{|}_{*}\;dx\to \int_{\Omega }\alpha |v{|}_{*}\;dx, \end{array}$$

which proves (5.6). ▪

**Lemma 5.3**

*Let* $\Omega \subset R^{N}$ *be a polyhedral domain and* $\alpha \in C(\bar{\Omega })$ *with* *α*(*x*)≥0 *in* *Ω*. *Let* (*w*_*h*_) *be a sequence that fulfils for all* *h*, *w*_*h*_∈*P*_1,*h*_(*Ω*)^*d*^ *and* |*w*_*h*_(*x*)|≤*α*(*x*) *for all* $x\in N_{h}$. *If* $w_{h}⇀w$ *for* *h*→0 *in* *L*^2^(*Ω*)^*d*^ *then it holds that* |*w*|≤*α* *a.e. in* *Ω*.

**Proof.**

The assertion follows by a slight modification of the proof of lemma 5.2. Instead of the piecewise constant interpolant we define *α*_*h*_ as the piecewise affine interpolant of *α*, i.e. *α*_*h*_=*I*_*h*_*α*, which fulfils *α*(*x*)=(*I*_*h*_*α*)(*x*) for all $x\in N_{h}$ and *α*_*h*_→*α* strongly in $L^{\infty }{\left(\Omega \right)}^{d}$. By (5.8), we obtain

$$\begin{array}{rl} \int_{\Omega }w\cdot v\;dx\leftarrow \int_{\Omega }w_{h}\cdot v_{h}\;dx & \;=\sum_{T\in T_{h}}\frac{\lambda (T)}{N+1}\sum_{x\in N_{h}\cap T}w_{h}(x)\cdot v_{h}{|}_{T}\;dx\\ & \;\leq \sum_{T\in T_{h}}\frac{\lambda (T)}{N+1}\sum_{x\in N_{h}\cap T}|w_{h}(x)||v_{h}{|}_{T}{|}_{*}\\ & \;\leq \sum_{T\in T_{h}}\frac{\lambda (T)}{N+1}\sum_{x\in N_{h}\cap T}\alpha (x)|v_{h}{|}_{T}{|}_{*}\\ & \;=\int_{\Omega }\alpha_{h}|v_{h}{|}_{*}\;dx\to \int_{\Omega }\alpha |v{|}_{*}\;dx. \end{array}$$

**Theorem 5.4**

*Let* $\Omega \subset R^{N}$ *be a polyhedral domain and* $\alpha \in C(\bar{\Omega })$ *such that (3.1) holds true. Then the sets
*

5.8 $$K_{h}=\{w\in P_{1,h}{\left(\Omega \right)}^{d}:|w(x_{T})|\leq \alpha (x_{T})\text{ for all }T\in T_{h}\}$$

*Mosco converge for h→0 to the set K(H*^1^*(Ω)*^*d*^*) in H*^1^*(Ω)*^*d*^.

**Proof.**

Since weak convergence in *H*^1^(*Ω*) implies weak convergence in *L*^2^(*Ω*), the preceding lemma 5.2 shows that (M1) is fulfilled. We now show (M2′). To prove the assertion, we may use a strategy that is similar to the one in [4], ch. II and requires (3.4). Note that theorem 3.1 implies that the set

5.9 $$\tilde{K}:=\{\varphi \in C^{\infty }{\left(\bar{\Omega }\right)}^{d}:|\varphi (x)|<\alpha (x)\text{ for all }x\in \bar{\Omega }\}$$

is also dense in *K*(*H*^1^(*Ω*)^*d*^) with respect to the *H*^1^(*Ω*)^*d*^-norm. For the global interpolation operator *I*_*h*_ defined in (5.3), we have the classical estimate,

5.10 $$∥u-I_{h}u{∥}_{L^{\infty }(\Omega )}\leq ch^{2}∥u{∥}_{W^{2,\infty }(\Omega )}\;\forall u\in W^{2,\infty }(\Omega ).$$

Here, *c* denotes a constant independent of *h* on account of the shape-regularity of the triangulation (5.2) (cf. [28, p.61]). We further define $r_{h}:\tilde{K}\to P_{1,h}{\left(\Omega \right)}^{d}$ by

$$r_{h}w:=[I_{h}w_{1},\ldots ,I_{h}w_{d}],$$

and it follows that *r*_*h*_*w*→*w* as *h*→0 in *H*^1^(*Ω*)^*d*^ for all $w\in \tilde{K}$; see [28, Corollary 1.109]. Applying estimate (5.11) to the components of $w\in \tilde{K}$ and using the equivalence of norms on $R^{d}$, one obtains that

5.11 $$∥\;|w-r_{h}w|\;{∥}_{L^{\infty }(\Omega )}\leq ch^{2}∥w{∥}_{W^{2,\infty }{\left(\Omega \right)}^{d}},$$

for a suitable modification of *c*. This implies

5.12 $$|r_{h}w(x)|\leq |w(x)|+ch^{2}∥w{∥}_{W^{2,\infty }{\left(\Omega \right)}^{d}}\;\forall x\in \Omega .$$

Since any $w\in \tilde{K}$ is uniformly bounded away from *α*, there exists *h*_0_=*h*_0_(*w*) such that *r*_*h*_*w*∈*K*_*h*_∀*h*≤*h*_0_, which implies (M2′). ▪

**Corollary 5.5**

*Under the conditions of theorem 5.4, the sequence* (*K*_*h*_) *defined in* (*5.8*) *Mosco converges for* *h*→0 *to the set* *K*(*L*^2^(*Ω*)^*d*^) *in* *L*^2^(*Ω*)^*d*^.

**Proof.**

Again, lemma 5.2 implies that (M1) with *X*=*L*^2^(*Ω*)^*d*^ holds true. For $\tilde{K}$ defined in (5.10) it holds that $\tilde{K}$ is also dense in *K*(*L*^2^(*Ω*)^*d*^) with respect to the *L*^2^(*Ω*)^*d*^-norm (cf. (3.2)). Thus, (M2′) follows analogously to the proof of theorem 5.4. ▪

**Corollary 5.6**

*Under the conditions of theorem* 5.4, *the node-based discrete sets*

5.13 $$K_{h}:=\{w\in P_{1,h}{\left(\Omega \right)}^{d}:|w(x)|\leq \alpha (x)\;\forall x\in N_{h}\},$$

*Mosco converge for* *h*→0 *to* *K*(*H*^1^(*Ω*)^*d*^) *in* *H*^1^(*Ω*)^*d*^.

**Proof.**

The proof is analogous to the proof of theorem 5.4, noting that (5.13) also implies *r*_*h*_*w*∈*K*_*h*_∀*h*≤*h*_0_ with *K*_*h*_ according to the node-based definition (5.14). ▪

**Remark 5.7**

With the help of the density property (3.2) for uniformly continuous upper bounds, the above results on the Mosco convergence of discretized convex sets carry over to spaces involving homogeneous Dirichlet boundary conditions. In this context, the set *P*_1,*h*_(*Ω*) in the definitions of the discretized sets *K*_*h*_ in (5.9) and (5.14) has to be replaced by the space

$$P_{1,h}^{\partial \Omega }:=\{u\in C(\bar{\Omega }):u{|}_{T}\in P_{1}\forall T\in T_{h},u(x)=0\;\forall x\in N_{h}\cap \partial \Omega \}.$$

The resulting discrete sets *K*_*h*_ incorporate the zero boundary condition and the corresponding results on Mosco convergence for *h*→0 remain valid replacing *H*^1^(*Ω*)^*d*^ by $H_{0}^{1}{\left(\Omega \right)}^{d}$.

With the help of the density result (3.2), one obtains the following result for the discrete approximation of pointwise constraint sets in *H*(div;*Ω*) by the Raviart–Thomas finite-element space *RT*_*h*_(*Ω*) (cf. (5.4)).

**Theorem 5.8**

*Let* $\Omega \subset R^{N}$ *be a polyhedral domain. Let* $\alpha \in C(\bar{\Omega })$ *such that (3.1) is satisfied. Then the sets
*

$$K_{h}:=\{w\in RT_{0,h}(\Omega ):|w(x_{T})|\leq \alpha (x_{T})\;\forall T\in T_{h}\}.$$

*Mosco converge to K(H*_0_*(div;Ω)) in H(div;Ω) and to K(L*^2^*(Ω)*^*N*^*) in L*^2^*(Ω)*^*N*^.

**Proof.**

Let *w*_*h*_∈*K*_*h*_ for all *h*. First observe that if (*w*_*h*_) weakly converges to *w* in *H*(div;*Ω*), then it also weakly converges to *w* in *L*^2^(*Ω*)^*N*^. Analogously to the proof of lemma 5.2 one concludes that |*w*|≤*α* a.e. in *Ω*. The continuity of the normal trace mapping

$$H(\mathrm{div};\Omega )∋w\mapsto {\left\langle w\nu ,v\right\rangle }_{H^{-1/2}(\partial \Omega ),H^{1/2}(\partial \Omega )}\in R$$

for fixed *v*∈*H*^1^(*Ω*) implies *wν*=0 in *H*^−1/2^(∂*Ω*). We conclude that *w*∈*K*(*H*_0_(div;*Ω*)) whence it follows that (M1) is satisfied. Secondly, note that

$${\bar{K(C_{c}^{\infty }{\left(\Omega \right)}^{N})}}^{H(\mathrm{div};\Omega )}=K(H_{0}(\mathrm{div};\Omega ));$$

cf. (3.2). For the global Raviart–Thomas interpolation operator defined in (5.5), the following interpolation error estimate holds true [28, Corollary 1.115]:

5.14 $$∥u-I_{h}^{\mathrm{RT}}u{∥}_{L^{\infty }{\left(\Omega \right)}^{N}}+∥\mathrm{div}\;u-\mathrm{div}\;I_{h}^{\mathrm{RT}}u{∥}_{L^{\infty }(\Omega )}\leq ch∥u{∥}_{W^{1,\infty }{\left(\Omega \right)}^{N}},$$

for all $u\in W^{2,\infty }{\left(\Omega \right)}^{N}$. Setting $r_{h}w:=I_{h}^{\mathrm{RT}}w$ for any $w\in \tilde{K}$, where

$$\tilde{K}:=\{w\in C_{c}^{\infty }{\left(\Omega \right)}^{N}:|w(x)|<\alpha (x),\;\forall x\in \Omega \},$$

and taking account of the fact that $I_{h}^{\mathrm{RT}}w\to w$ in *H*(div) for all $w\in \tilde{K}$, we may proceed analogously to the proof of theorem 5.4 to verify (M2′). ▪

The previous approach can also be applied to derive approximation results for constraint sets involving pointwise bounds on partial derivatives. To begin with, we consider the gradient-constraint sets

$$K_{\nabla }(X(\Omega ))=\{w\in X(\Omega ):|\nabla w|\leq \alpha \text{ a.e. in }\Omega \},$$

for *X*(*Ω*)⊂*H*^1^(*Ω*)^*d*^ and an arbitrary norm |⋅| on $R^{N\times d}$.

**Theorem 5.9**

*Let* $\Omega \subset R^{N}$ *be a polyhedral domain. Let* $\alpha \in C(\bar{\Omega })$ *such that (3.1) is satisfied. Define
*

5.15 $$K_{h}:=\{w\in P_{1,h}^{\partial \Omega }{\left(\Omega \right)}^{d}:|\nabla w{|}_{T}|\leq \alpha (x_{T})\;\forall T\in T_{h}\}.$$

*Then the sets K*_*h*_ *Mosco converge to* $K_{\nabla }(H_{0}^{1}{\left(\Omega \right)}^{d})$ *in* $H_{0}^{1}{\left(\Omega \right)}^{d}$.

**Proof.**

To prove (M1), it suffices to notice that if $w_{h}⇀w$ in *H*^1^(*Ω*)^*d*^ then $\nabla w_{h}⇀\nabla w$ in *L*^2^(*Ω*)^*N*×*d*^. Similar to the proof of lemma 5.2, one obtains for *v*∈*C*_*c*_(*Ω*)^*N*×*d*^ that

$$\int_{\Omega }\nabla w:v\;dx\leftarrow \int_{\Omega }\nabla w_{h}:v\;dx\leq \int_{\Omega }|\nabla w_{h}||v{|}_{*}\;dx\leq \int_{\Omega }\alpha_{h}|v{|}_{*}\;dx\to \int_{\Omega }\alpha |v{|}_{*}\;dx,$$

using *α*_*h*_ from (5.7). Therefore, (5.6) holds with ∇*w* in place of *w*, and (M1) is verified.

To prove (M2′), we consider again the global interpolation operator *I*_*h*_ from (5.3). The standard estimate

$$∥\nabla u-\nabla I_{h}u{∥}_{L^{\infty }{\left(\Omega \right)}^{N}}\leq ch∥u{∥}_{W^{2,\infty }(\Omega )},\;\forall u\in W^{2,\infty }(\Omega ),$$

holds true (e.g. [28]). Note also that $K_{\nabla }(C_{c}^{\infty }{\left(\Omega \right)}^{d})$ is dense in $K_{\nabla }(H_{0}^{1}{\left(\Omega \right)}^{d})$ for the *H*^1^(*Ω*)^*d*^-norm [11, Theorem 4]. Thus, the set

$$\tilde{K}:=\{w\in C_{c}^{\infty }{\left(\Omega \right)}^{d}:|\nabla w(x)|<\alpha (x)\;\forall x\in \Omega \}$$

is also dense in *K*_∇_(*H*^1^(*Ω*)^*d*^). Therefore, one may argue as in the proof of theorem 5.4 to deduce (M2′). ▪

Next we consider pointwise constraints on the divergence. For *X*(*Ω*)⊂*H*(div;*Ω*) let

5.16 $$K_{\mathrm{div}}(X(\Omega )):=\{w\in X(\Omega ):|\mathrm{div}\;w|\leq \alpha \text{ a.e. in }\Omega \}.$$

Using Raviart–Thomas finite elements, a discrete realization of the inequality constraint in (5.17) can be achieved by imposing the inequality on the midpoints of the triangulation. The following statement ensures that the resulting approach is stable as the mesh width goes to zero.

**Theorem 5.10**

*Let* $\Omega \subset R^{N}$ *be a polyhedral domain. Let* $\alpha \in C(\bar{\Omega })$ *fulfil (3.1). Then the sets
*

$$K_{h}:=\{w\in RT_{0,h}(\Omega ):|\mathrm{div}\;w{|}_{T}|\leq \alpha (x_{T}),\forall T\in T_{h}\}$$

*Mosco converge in H(div;Ω) to the set K*_div_*(H*_0_*(div;Ω)) as defined in (*5.17*).*

**Proof.**

Taking account of the fact that $w_{h}⇀w$ in *H*(div;*Ω*), *w*_*h*_∈*K*_*h*_, implies $\mathrm{div}\;w_{h}⇀\mathrm{div}\;w$ in *L*^2^(*Ω*), (M1) follows analogously to the corresponding part of the proof of corollary 5.9. Since $K_{\mathrm{div}}(C_{c}^{\infty }{\left(\Omega \right)}^{N})$ is dense in *K*_div_(*H*_0_(div;*Ω*)) [11, Theorem 4], the set

$$\tilde{K}:=\{w\in C_{c}^{\infty }{\left(\Omega \right)}^{d}:|\mathrm{div}w(x)|<\alpha (x),\forall x\in \Omega \}$$

is also dense in *K*_div_(*H*_0_(div;*Ω*)). Setting $r_{h}=I_{h}^{\mathrm{RT}}$, the estimate (5.15) implies *r*_*h*_*w*→*w* in *H*(div;*Ω*) and

$$∥\mathrm{div}\;w-\mathrm{div}\;r_{h}w{∥}_{L^{\infty }(\Omega )}\leq ch∥w{∥}_{W^{2,\infty }{\left(\Omega \right)}^{N}},$$

for all *w* in $\tilde{K}$. In particular, one may argue as in the proof of theorem 5.4 to verify (M2′). ▪

For a general *L*^*p*^-function as upper bound, a point-based discretization is obviously not possible. As a remedy, the construction of the discrete sets *K*_*h*_ typically involves some kind of averaging process. For this purpose, we define the integral mean

$${⨍}_{T}\alpha \;dx:=\frac{\int_{T}\alpha \;dx}{\lambda (T)}$$

over some given subset *T*⊂*Ω* (with positive measure).

Now we have to take into account that the density results of the type (3.2) and (3.4), which represent the main ingredient to prove the consistency of the finite-element approximation, may fail to hold true (e.g. theorem 4.2). On the other hand, the results from §4 indicate that the density property is still guaranteed for a large class of discontinuous obstacles. To maintain the greatest level of generality, we assume that the non-negative measurable function α:Ω→R∪{+∞} allows for the density property

5.17 $${\bar{K(C(\bar{\Omega }))}}^{L^{2}{\left(\Omega \right)}^{d}}=K(L^{2}{\left(\Omega \right)}^{d}).$$

Here, we concentrate on the consistency in the *L*^2^-topology but an extension to the other cases is possible by appropriately modifying assumption (5.18). We stress the fact that assumption (5.18) is fulfilled in relevant situations (cf. e.g. theorem 4.4).

**Lemma 5.11**

*Let* $\Omega \subset R^{N}$ *be a polyhedral domain and* *α*∈*L*^2^(*Ω*) *with* *α*(*x*)≥0 *a.e. in* *Ω*. *Let* (*w*_*h*_) *be a sequence that fulfils for all* *h*, *w*_*h*_∈*P*_1,*h*_(*Ω*)^*d*^ *and* |*w*_*h*_(*x*_*T*_)|≤ $|w_{h}(x_{T})|\leq {⨍}_{T}\alpha \;dx$ *for all* $T\in T_{h}$. *If* $w_{h}⇀w$ *for* *h*→0 *in* *L*^2^(*Ω*)^*d*^ *then it holds that* |*w*|≤*α* *a.e. in* *Ω*.

**Proof.**

The assertion follows analogously to the proof of lemma 5.2 by a slight modification of the definition of *α*_*h*_. Instead of the piecewise constant interpolant we consider the piecewise constant quasi-interpolant $\alpha_{h}:=\sum_{T\in T_{h}}\chi_{T}{⨍}_{T}\alpha \;dx$. Observe that *α*_*h*_ converges strongly to *α* in *L*^2^(*Ω*)^*d*^, which is sufficient for the above argument. ▪

**Theorem 5.12**

*Let* $\Omega \subset R^{N}$ *be a polyhedral domain. Let α∈L*^2^*(Ω) with (3.1) such that (5.17) holds true. Then the sets
*

$$K_{h}:=\left\{w\in P_{1,h}{\left(\Omega \right)}^{d}:|w(x_{T})|\leq {⨍}_{T}\alpha \;dx,\forall T\in T_{h}\right\}.$$

*Mosco converge for h→0 to the set K(L*^2^*(Ω)*^*d*^*) in L*^2^*(Ω)*^*d*^.

**Proof.**

We only need to prove (M2′) since corollary 5.11 implies (M1). First note that (3.1) and (5.18) imply that the set

$$\tilde{K}:=\{w\in C_{c}^{\infty }{\left(\Omega \right)}^{d}:\;\exists \;\delta =\delta (w)>0\text{ such that }|w(x)|\leq \alpha (x)-\delta \text{ a.e. in }\Omega \},$$

is also dense in *K*(*L*^2^(*Ω*)^*d*^). Furthermore, we set

$$r_{h}w:=[I_{h}w_{1},\ldots ,I_{h}w_{d}],$$

for $w\in \tilde{K}$ and *I*_*h*_ as in (5.3). Integrating estimate (5.13) yields

$$\left|{⨍}_{T}r_{h}w\;dx\right|\leq {⨍}_{T}|w|\;dx+ch^{2}∥w{∥}_{W^{2,\infty }{\left(\Omega \right)}^{d}},\;\forall T\in T_{h}.$$

Let $w\in \tilde{K}$ be fixed. Since *r*_*h*_*w* is affine on each $T\in T_{h}$, an application of the midpoint rule shows

$$|r_{h}w(x_{T})|\leq {⨍}_{T}|w|\;dx+ch^{2}∥w{∥}_{W^{2,\infty }{\left(\Omega \right)}^{d}},\;\forall T\in T_{h},$$

which implies

5.18 $$|r_{h}w(x_{T})|\leq {⨍}_{T}\alpha \;dx-\delta (w)+ch^{2}∥w{∥}_{W^{2,\infty }{\left(\Omega \right)}^{d}},\;\forall T\in T_{h}.$$

This implies *r*_*h*_*w*∈*K*_*h*_ for all $w\in \tilde{K}$ and *h*≤*h*_0_(*w*). By (5.11), it holds that *r*_*h*_*w*→*w* in *L*^2^(*Ω*)^*d*^ for *h*→0, which proves (M2′). ▪

## 6. Further applications

### (a) Regularization of elasto-plastic contact problems

In the context of the one time-step problem of quasi-static elasto-plasticity with an associative flow law, the deformation of a material represented by a bounded Lipschitz domain *Ω* subject to given applied forces is modelled by the evolution of the displacement, the material stress and strain as well as certain internal variables (cf. [6]). An elasto-plastic contact problem arises if the movement of the material is additionally restricted by the presence of a rigid obstacle. From a mathematical point of view, the problem can be equivalently reformulated in terms of the normal stress z* at the (sufficiently smooth) contact boundary *Γ*_*c*_⊂∂*Ω*, and a variable *q* that is related to the deviatoric part of the material stress; for details we refer to [24, p.154]:

6.1 $$\begin{array}{rl} \min\; & G([z^{*},q])-\langle z^{*},\psi \rangle \;\text{over }[z^{*},q]\in H^{1/2}{\left(\Gamma_{c}\right)}^{*}\times L^{2}{\left(\Omega \right)}^{d}\\ \text{s.t.}\; & z^{*}\in H_{+}^{1/2}{\left(\Gamma_{c}\right)}^{*},\\ & \;|q{|}_{2}\leq \beta \text{ a.e. in }\Omega . \end{array}\}$$

Here, *d*:=*N*(*N*+1)/2−1 and *G* is a strongly convex, continuous and coercive functional that models the elasto-plastic material behaviour subject to given external loads. Furthermore, a contact constraint on the normal component of the displacement is imposed by a function *ψ*, which lies in the trace space *H*^1/2^(*Γ*_*c*_). The upper bound *β*∈*L*^2^(*Ω*) is determined by the hardening law, and it is bounded away from zero by the positive yield stress *σ*_*y*_, i.e. *β*(*x*)≥*σ*_*y*_ a.e. in *Ω*. The normal stress z* is constrained to lie in the polar cone

$$H_{+}^{1/2}{\left(\Gamma_{c}\right)}^{*}:=\{z^{*}\in H^{1/2}{\left(\Gamma_{c}\right)}^{*}:\langle z^{*},z\rangle \leq 0\;\forall z\in H_{+}^{1/2}(\Gamma_{c})\}$$

to the cone of non-negative functions

$$H_{+}^{1/2}(\Gamma_{c})=\{z\in H^{1/2}(\Gamma_{c}):z\geq 0\text{ a.e. }\text{on }\Gamma_{c}\},$$

where *H*^1/2^(*Γ*_*c*_)* designates the topological dual space of *H*^1/2^(*Γ*_*c*_). From an algorithmic point of view, it is favourable to replace (6.1) by a combined Moreau–Yosida/Tikhonov regularization given by

6.1*γ* $$\begin{array}{cc} \; & \begin{array}{rl} \min\; & G([z,q])-{\left(z,\psi \right)}_{L^{2}(\Gamma_{c})}+\frac{\gamma_{n}}{2}∥z^{+}{∥}_{L^{2}\left(\Gamma_{c}\right)}^{2}\\ & \;\;+\frac{\gamma_{n}}{2}∥{\left[(|q{|}_{2}-\beta )\right]}^{+}{∥}_{L^{2}(\Omega )}^{2}+\frac{1}{2\gamma_{n}^{\prime }}∥[z,q]{∥}_{H^{1}(\Gamma_{c})\times H^{1}{\left(\Omega \right)}^{d}}^{2},\\ \text{over}\; & \;[z,q]\in H^{1}(\Gamma_{c})\times H^{1}{\left(\Omega \right)}^{d}, \end{array}\} \end{array}$$

where (*γ*_*n*_) and (*γ*_*n*_′) are sequences with $\gamma_{n},\gamma_{n}^{\prime }\to +\infty$ as n→+∞. In contrast to (6.1), (6.2) can be solved efficiently by the semismooth Newton method in the infinite-dimensional setting. As a consequence, the Newton iterates are superlinearly convergent, and the convergence rate is mesh-independent upon discretization. For details, see [24, section 5]. In order to prove the stability of (6.2) with regard to the limit problem (6.1) in the sense of theorem 2.1, we show that the problems (6.2) define a quasi-monotone perturbation of $i_{K}$ with respect to the dense subspace *H*^1^(*Γ*_*c*_)×*H*^1^(*Ω*)^*d*^⊂*H*^1/2^(*Γ*_*c*_)*×*L*^2^(*Ω*)^*d*^ (cf. definition (2.2)). Here, we write for $X\subset H^{1/2}{\left(\Gamma_{c}\right)}^{*}\times L^{2}{\left(\Omega \right)}^{d}$,

$$K(X):=\{[z^{*},q]\in X:z^{*}\in H_{+}^{1/2}{\left(\Gamma_{c}\right)}^{*},\;|q{|}_{2}\leq \beta \text{ a.e. in }\Omega \},$$

and $K:=K(H^{1/2}{\left(\Gamma_{c}\right)}^{*}\times L^{2}{\left(\Omega \right)}^{d})$. In fact, setting

$$R_{n}([z,q]):=\frac{\gamma_{n}}{2}∥z^{+}{∥}_{L^{2}\left(\Gamma_{c}\right)}^{2}+\frac{\gamma_{n}}{2}∥{\left[(|q{|}_{2}-\beta )\right]}^{+}{∥}_{L^{2}(\Omega )}^{2}+\frac{1}{2\gamma_{n}^{\prime }}∥[z,q]{∥}_{H^{1}(\Gamma_{c})\times H^{1}{\left(\Omega \right)}^{d}}^{2},$$

where it is understood that $R_{n}([z^{*},q])=+\infty$, unless [z*,*q*]∈*H*^1^(*Γ*_*c*_)×*H*^1^(*Ω*)^*d*^, it is easily seen that

$${\bar{R}}_{n}([z,q]):=i_{K}([z,q])+\frac{1}{2\gamma_{n}^{\prime }}∥[z,q]{∥}_{H^{1}(\Gamma_{c})\times H^{1}{\left(\Omega \right)}^{d}}^{2},$$

fulfils (2.4). Moreover, we set

$${\underline{R}}_{n}([z^{*},q]):=\frac{\gamma_{n}}{2}r(z^{*})+\frac{\gamma_{n}}{2}∥{\left[(|q{|}_{2}-\beta )\right]}^{+}{∥}_{L^{2}(\Omega )}^{2},$$

where

$$r(z^{*}):={\left(\max\left\{\underset{\begin{array}{c} z\in H_{+}^{1/2}(\Gamma_{c}),\\ ∥z{∥}_{H^{1/2}(\Gamma_{c})}=1 \end{array}}{\sup}\langle z^{*},z\rangle ,0\right\}\right)}^{2}.$$

The validity of (2.3) is an immediate consequence of the following lemma.

**Lemma 6.1**

*The functional* $r:H^{1/2}{\left(\Gamma_{c}\right)}^{*}\to R$ *is weakly l.s.c. and it fulfils*

(*i*) *r*(z*)=0 *for all* z*∈*H*^1/2^_+_(*Γ*_*c*_)*,
(*ii*) *r*(z*)>0 *for all* z*∈*H*^1/2^(*Γ*_*c*_)*∖*H*^1/2^_+_(*Γ*_*c*_)*,
(*iii*) $r(z)\leq {∥z^{+}∥}_{L^{2}(\Gamma_{c})}^{2}$ *for all* *z*∈*L*^2^(*Γ*_*c*_).

**Proof.**

As a composition of a convex, continuous and monotone function with a supremum of l.s.c. and convex functions, $r:H^{1/2}{\left(\Gamma_{c}\right)}^{*}\to R$ is weakly l.s.c. Assertions (i) and (ii) are direct consequences of the definition of $H_{+}^{1/2}{\left(\Gamma_{c}\right)}^{*}$. For *z*∈*L*^2^_−_(*Γ*_*c*_)={*z*∈*L*^2^(*Γ*_*c*_):*z*≤ 0 a.e. in *Ω*}, it holds *r*(*z*)=0 and (iii) is always satisfied. Now let *z*∈*L*^2^(*Γ*_*c*_)∖*L*^2^_−_(*Γ*_*c*_). By the density of *H*^1/2^_+_(*Γ*_*c*_) in *L*^2^_+_(*Γ*_*c*_) it holds that

$$r{\left(z\right)}^{1/2}=\underset{\begin{array}{c} \tilde{z}\in H_{+}^{1/2}(\Gamma_{c})\\ ∥\tilde{z}{∥}_{H^{1/2}(\Gamma_{c})}=1 \end{array}}{\sup}\langle z,\tilde{z}\rangle >0.$$

Moreover, one obtains

$$\begin{array}{rl} & \;∥z^{+}{∥}_{L^{2}(\Gamma_{c})}=\underset{\begin{array}{c} \tilde{z}\in L^{2}(\Gamma_{c})\\ \tilde{z}\neq 0 \end{array}}{\sup}\frac{1}{∥\tilde{z}{∥}_{L^{2}(\Gamma_{c})}}(z^{+},\tilde{z})\\ & \;\;\geq \underset{\begin{array}{c} \tilde{z}\in L^{2}(\Gamma_{c})\\ \tilde{z}\neq 0,\tilde{z}\geq 0\text{ a.e. } \end{array}}{\sup}\frac{1}{∥\tilde{z}{∥}_{L^{2}(\Gamma_{c})}}(z,\tilde{z})\geq \underset{\begin{array}{c} \tilde{z}\in H_{+}^{1/2}(\Gamma_{c})\\ \tilde{z}\neq 0 \end{array}}{\sup}\frac{1}{∥\tilde{z}{∥}_{H^{1/2}(\Gamma_{c})}}(z,\tilde{z})=r{\left(z\right)}^{1/2}, \end{array}$$

which implies (iii). ▪

From the discussion in the introduction and theorem 2.1, it is known that the consistency of the regularization scheme (6.2) with respect to (6.1) hinges on the density of *K*(*H*^1^(*Ω*)^*d*^) in *K*(*L*^2^(*Ω*)^*d*^), where

$$K(X(\Omega )):=\{q\in X(\Omega ):|q{|}_{2}\leq \beta \text{ a.e. in }\Omega \},$$

in accordance with the notation from the preceding sections. Owing to the results of §§3 and 4, this is always fulfilled for kinematic hardening, as *β* is a positive constant in this case. In the same way, it is also fulfilled for large classes of discontinuous obstacles *β* in the case of combined isotropic-kinematic hardening. Once the density property is ensured, one may use monotonicity properties of *G* to derive strong convergence properties of regularized (normal) stresses, strains and displacement; cf. [24] for details.

### (b) Fenchel duality in image restoration

Optimization problems with total variation regularization have been successfully considered in the image restoration context. In the denoizing setting, an original image *u*_true_ that belongs to the space of functions of bounded variation *BV*(*Ω*), $\Omega \subset R^{2}$, is sought to be recovered from a noise perturbed measurement *f*=*u*_true_+*η* with *η*∈*L*^2^(*Ω*), ∫η=0 and $\int |\eta {|}^{2}=\sigma^{2}$. This motivates the optimization problem

$$\text{min}\;\frac{1}{2}\int_{\Omega }|u-f{|}^{2}\;dx+\alpha \int_{\Omega }|Du{|}_{1}\;\text{over }u\in \mathrm{BV}(\Omega ),$$

for α∈R in the seminal work by Rudin *et al*. [31]. Here, Du, the distributional gradient of *u*∈*BV*(*Ω*), is a Borel measure and $|Du{|}_{1}$ is its total variation measure with total mass $\int_{\Omega }|Du{|}_{1}$, which is characterized via duality as

$$\int_{\Omega }|Du{|}_{1}=\sup\left\{\int_{\Omega }u\mathrm{div}v\;dx:v\in C_{c}^{1}(\Omega ;R^{2}),\;|v(x){|}_{\infty }\leq 1,\forall x\in \Omega \right\}.$$

The drawback of the above reconstruction scheme is that the choice of the regularization parameter *α* is global: A good reconstruction locally requires high values of *α* in some regions of *Ω* (e.g. flat regions of *u*_true_) and low values in other regions (e.g. locations of details of *u*_true_). A recent approach in [32,33] proposes the following alternative: For a function α:Ω→R such that (3.1) holds true, consider the optimization problem

6.2 $$\min\;\frac{1}{2}\int_{\Omega }|u-f{|}^{2}\;dx+\int_{\Omega }\alpha (x)|Du{|}_{1}\;\text{over }u\in \mathrm{BV}(\Omega ),$$

where $\int_{\Omega }\alpha (x)|Du{|}_{1}$ stands for the integral of *α* on *Ω* with respect to the measure $|Du{|}_{1}$. Hence, *α* needs to be a $|Du{|}_{1}$-integrable function in order for $\int_{\Omega }\alpha |Du{|}_{1}$ to be correctly defined. A sufficient condition for this is given by *α*∈*C*(*Ω*), the space of continuous functions on *Ω*.

As usual in convex optimization, it is convenient to consider the problem in (6.3) from the point of view of Fenchel duality. In fact, (6.3) can be characterized as the Fenchel dual problem of the following constrained optimization problem:

6.3 $$\begin{array}{rl} \min\; & \frac{1}{2}∥\mathrm{div}\;p+f{∥}_{L^{2}(\Omega )}^{2}\;\text{over }p\in H_{0}(\mathrm{div};\Omega )\\ \text{s.t.}\; & p\in K(H_{0}(\mathrm{div};\Omega ),|\cdot {|}_{\infty }), \end{array}\}$$

if the following density result holds true:

$${\bar{K(C_{c}^{1}{\left(\Omega \right)}^{2}),|\cdot {|}_{\infty })}}^{H_{0}(\mathrm{div};\Omega )}=K(H_{0}(\mathrm{div};\Omega ),|\cdot {|}_{\infty }),$$

where, according to the above notational convention,

$$K(H_{0}(\mathrm{div};\Omega ),|\cdot {|}_{\infty })=\{q\in H_{0}(\mathrm{div};\Omega ):|q(x){|}_{\infty }\leq \alpha (x)\text{ a.e. in }\Omega \}.$$

## 7. Conclusion

We investigate the stability of a large number of perturbation and dualization approaches to variational inequality and constrained optimization problems in the context of density properties of a convex constraint set. If the intersection with certain dense subspaces is dense in the feasible set, one may prove the unconditional consistency of various perturbation schemes including Galerkin approximations. In this regard, the class of quasi-monotone perturbations provides a unified framework.

The abstract motivation leads to the study of density properties of constraint sets in Sobolev spaces with respect to spaces of smooth functions. We focus specifically on sets that are defined by a pointwise constraint on the norm of the function value. In this case, the density property is determined by the regularity of the upper bound. Whereas the case of a uniformly continuous obstacle gives rise to positive density results in various Sobolev spaces, the result fails to be valid in general, if the obstacle is just a Sobolev function. However, a large variety of discontinuous upper bounds still remains compatible with the density property. This includes functions that fulfil a generalized lower semicontinuity condition as well as supersolutions of elliptic PDEs.

Density results further allow to deduce the Mosco convergence of various finite-element discretized constraint sets in Sobolev spaces. Finally, the previous results are applied in the context of the regularization of quasi-static elasto-plastic contact problems and the dualization of total variation-based image restoration problems.

Our future research is concerned with the refined characterization of the class of upper bounds that comply with the density property. Another interesting direction of future research, which we plan to pursue, is related to constraint qualifications (CQs) in the context of Fenchel–Legendre dualization in convex and possibly non-smooth optimization. Here it appears that the density of convex intersections may provide a suitable constraint qualification implying duality without a duality gap. Such a density CQ appears to neither imply nor be contained in currently known constraint qualifications like those used in the work by Hedy Attouch and co-authors (e.g. [34]). Another current research aspect concerns the ramification of remark 3.2(ii). In the presence of an inhomogeneous Dirichlet boundary condition, the construction of suitable trace-preserving mollification operators with variable support appears promising. Those operators are also of utmost interest in the context of non-smooth variational problems in image restoration.

## Data accessibility

This paper has no additional data.

## Appendix A. Properties of quasi-monotone perturbations

Proof of theorem 2.1 Denote by

$$\Gamma \text{-}\underset{n\to +\infty }{lim sup}G_{n}(u):=\underset{U\in N(u)}{\sup}\underset{n\to +\infty }{lim sup}\underset{u\in U}{\inf}G_{n}(u),$$

the *Γ*-upper limit at *u* of a sequence of functions $G_{n}:X\to R\cup \{+\infty \}$ in the norm topology. Here, N(u) denotes the set of all open neighbourhoods of *u* in the norm of *X*. By analogy, define $\Gamma_{w}\text{-}\underset{n\to +\infty }{lim sup}G_{n}$, the *Γ*-upper limit of *G*_*n*_ in the weak topology of *X*, as well as the lower limit counterpart $\Gamma_{w}\text{-}\underset{n\to +\infty }{lim inf}G_{n}$. We write

$$\Gamma_{w}\text{-}\lim_{n\to +\infty }G_{n}=\Gamma_{w}\text{-}\underset{n\to +\infty }{lim sup}G_{n}=\Gamma_{w}\text{-}\underset{n\to +\infty }{lim inf}G_{n},$$

for the weak *Γ*-limit of (*G*_*n*_) provided the latter equality is satisfied. For the corresponding definitions we refer to the monograph [12]. Further denote by *sc*^−^*G* the lower semicontinuous envelope of G:X→R∪{+∞}. Exploiting the relations between *Γ*- and pointwise convergence [12, ch. 5], one obtains with (2.4) and the continuity of *F*,

$$\begin{array}{rl} \Gamma_{w}\text{-}\underset{n\to +\infty }{lim sup}(F+R_{n}) & \;\leq \Gamma \text{-}\underset{n\to +\infty }{lim sup}(F+R_{n})\\ & \;\leq \Gamma \text{-}\underset{n\to +\infty }{lim sup}(F+{\bar{R}}_{n})={\mathrm{sc}}^{-}(F+i_{K\cap Y})=F+i_{\bar{K\cap Y}}, \end{array}$$

where we use [12, Prop. 6.3, Prop. 6.7, Prop. 5.7, Prop. 3.7]. Similarly, (2.3) together with [12, Prop. 6.7, Prop. 5.4] implies

A 1 $$\Gamma_{w}\text{-}\underset{n\to +\infty }{lim inf}(F+R_{n})\geq \Gamma_{w}\text{-}\underset{n\to +\infty }{lim inf}(F+{\underline{R}}_{n})=\lim_{n\to +\infty }{\mathrm{sc}}_{w}^{-}(F+{\underline{R}}_{n}),$$

where ${\mathrm{sc}}_{w}^{-}(F+{\underline{R}}_{n})$ denotes the lower semicontinuous envelope of $F+{\underline{R}}_{n}$ in the weak topology of *X*. Further note that the coercivity and the sequential weak lower semicontinuity of $F+{\underline{R}}_{n}$ imply that the level sets {*u*∈*X*:*F*(*u*)+*R*_*n*_(*u*)≤*t*}, t∈R, are bounded and sequentially weakly closed. If *X* is reflexive or if the dual space *X** is separable, then the sequential weak closure of bounded subsets of *X* coincides with the weak closure, see [12], Prop. 8.7, Prop. 8.14, such that $F+{\underline{R}}_{n}$ is weakly lower semicontinuous, which entails

$$\Gamma_{w}\text{-}\underset{n\to +\infty }{lim inf}(F+R_{n})\geq \lim_{n\to +\infty }(F+{\underline{R}}_{n})=F+i_{K},$$

by (A 1). Eventually, it holds that

$$\begin{array}{rl} F+i_{K} & \;\leq \Gamma_{w}\text{-}\underset{n\to +\infty }{lim inf}(F+R_{n})\\ & \;\leq \Gamma_{w}\text{-}\underset{n\to +\infty }{lim sup}(F+R_{n})\leq \Gamma \text{-}\underset{n\to +\infty }{lim sup}(F+R_{n})\leq F+i_{\bar{K\cap Y}}, \end{array}$$

such that $\Gamma \text{-}\lim_{n\to +\infty }(F+R_{n})=\Gamma_{w}\text{-}\lim_{n\to +\infty }(F+R_{n}u)=F+i_{K}$, if (1.1) holds true. ▪

Proof of proposition 2.6 Let $x\in K\setminus \bar{K\cap Y}$ and *ρ*>0 such that $\bar{B_{\rho }(x)}\cap \bar{K\cap Y}=\emptyset$, where *B*_*ρ*_(*x*):={*y*∈*X*:∥*x*−*y*∥<*ρ*}. (a) We first prove the following result:

A 2 $$\forall n\in N\;\exists \;\gamma_{n}>0:\;\left[y\in X\wedge \mathrm{dist}{\left(y,K\cap \bar{B_{\rho }(x)}\right)}^{2}<\frac{1}{\gamma_{n}}⟹y\notin X_{n}\right].$$

Assume that the opposite holds, i.e.

$$\;\exists \;n_{0}\in N:\;\left[\forall n\in N\;\exists \;x_{n}\in X_{n_{0}},v_{n}\in K\cap \bar{B_{\rho }(x)}:\;∥x_{n}-v_{n}{∥}^{2}\leq \frac{1}{n}\right].$$

Since $v_{n}\in \bar{B_{\rho }(x)}\cap K$ for all n∈N and $\bar{B_{\rho }(x)}\cap K$ is convex, bounded and closed, there exists a subsequence (*v*_*n*_*k*__) of (*v*_*n*_) with $v_{n_{k}}⇀v$ and $v\in \bar{B_{\rho }(x)}\cap K$. As *x*_*n*_−*v*_*n*_→0, one also obtains $x_{n_{k}}⇀v$ and thus *v*∈*X*_*n*_0__. Hence, $v\in X_{n_{0}}\cap K\cap \bar{B_{\rho }(x)}=\emptyset$, which is a contradiction. (b) Non-existence of a strong recovery sequence: Choose (*γ*_*n*_) according to (7.2) and assume that there exists a recovery sequence (*y*_*n*_) to *x*, which means that *y*_*n*_→*x* and *F*(*y*_*n*_)+(*γ*_*n*_/2)dist(*y*_*n*_,*K*)^2^+*i*_*X*_*n*__(*y*_*n*_)→*F*(*x*). The continuity of *F* implies that *y*_*n*_∈*X*_*n*_ for sufficiently large *n* and that (*γ*_*n*_/2)dist(*y*_*n*_,*K*)^2^→0. Consequently, using *y*_*n*_→*x* and *x*∈*K*, there exists $n_{1}\in N$ such that

$$\mathrm{dist}{\left(y_{n},K\right)}^{2}=\mathrm{dist}{\left(y_{n},K\cap B_{\rho }(x)\right)}^{2}\leq \frac{1}{\gamma_{n}},$$

for all *n*≥*n*_1_. With the help of part (*a*), we conclude that *y*_*n*_∉*X*_*n*_ for all *n*≥*n*_1_, which is a contradiction. ▪
